# Potential Environmental and Health Implications from the Scaled-Up Production and Disposal of Nanomaterials Used in Biosensors

**DOI:** 10.3390/bios12121082

**Published:** 2022-11-25

**Authors:** Kelli M. McCourt, Jarad Cochran, Sabah M. Abdelbasir, Elizabeth R. Carraway, Tzuen-Rong J. Tzeng, Olga V. Tsyusko, Diana C. Vanegas

**Affiliations:** 1 Department of Environmental Engineering and Earth Sciences, Clemson University, Clemson, SC 29634, USA; 2 Global Alliance for Rapid Diagnostics (GARD), Michigan State University, East Lancing, MI 48824, USA; 3 Department of Plant and Soil Sciences, University of Kentucky, Lexington, KY 40546, USA; 4 Central Metallurgical Research and Development Institute, P.O. Box 87, Helwan 11421, Egypt; 5 Department of Biological Sciences, Clemson University, Clemson, SC 29634, USA; 6 Interdisciplinary Group for Biotechnology Innovation and Ecosocial Change (BioNovo), Universidad del Valle, Cali 76001, Colombia

**Keywords:** concentration, dimensionality, life cycle, nanocomposite, nanomaterial, nanotoxicity, surface chemistry, toxicity, transformation

## Abstract

Biosensors often combine biological recognition elements with nanomaterials of varying compositions and dimensions to facilitate or enhance the operating mechanism of the device. While incorporating nanomaterials is beneficial to developing high-performance biosensors, at the stages of scale-up and disposal, it may lead to the unmanaged release of toxic nanomaterials. Here we attempt to foster connections between the domains of biosensors development and human and environmental toxicology to encourage a holistic approach to the development and scale-up of biosensors. We begin by exploring the toxicity of nanomaterials commonly used in biosensor design. From our analysis, we introduce five factors with a role in nanotoxicity that should be considered at the biosensor development stages to better manage toxicity. Finally, we contextualize the discussion by presenting the relevant stages and routes of exposure in the biosensor life cycle. Our review found little consensus on how the factors presented govern nanomaterial toxicity, especially in composite and alloyed nanomaterials. To bridge the current gap in understanding and mitigate the risks of uncontrolled nanomaterial release, we advocate for greater collaboration through a precautionary One Health approach to future development and a movement towards a circular approach to biosensor use and disposal.

## 1. Introduction

Nanoparticles are naturally occurring or engineered particles or materials less than 100 nm in size in at least one dimension. According to the European Commission a Nanomaterial contains 50% or more particles in that size range [1]. At the nanoscale, particles often exist with unique physical properties distinct from the properties of their bulk form due to increased relative surface area per volume unit and the dominance of quantum effects [2,3]. The enhanced optical, magnetic, electrical, and catalytic properties of nanomaterials, including nanometals, nanometal oxides, carbon-based nanomaterials, and inorganic two-dimensional nanoparticles, have made them useful in various applications. Specifically, the field of nano biosensing has significantly expanded the frontiers for the development of high-performance sensing devices [4,5].

Biosensors employ biological or biomimetic recognition elements (e.g., antibodies, enzymes, molecularly imprinted polymers, aptamers, etc.) to selectively bind (and, in some cases, react) with target analytes potentially present in complex samples. Biosensor applications span a wide spectrum including the fields of medicine, food safety, drug development, and environmental monitoring [6]. The biological elements typically utilized in biosensing platforms are often combined with nanomaterials with different compositions and dimensions [7] to facilitate or enhance the recognition and transduction processes of the operating mechanism of the biosensor device [8,9,10]. Generally, operation modes include electrochemical (measures changes in voltage, current, capacitance, impedance, etc.), electronic, optical (measures changes in fluorescence, luminescence, optical diffraction, etc.), piezoelectric, thermometric, acoustic, and colorimetric detection mechanisms.

Currently, nanoparticle-based biosensors are produced and utilized at different scales ranging from laboratory to industrial domains [11]. In most cases, nanomaterial-enabled technologies, such as biosensors and bioelectronics, are manufactured, used, and disposed of in a linear *end-of-life* fashion. Since the existing research on fate, transport, transformations, bioaccumulation, biomagnification, and toxicity of engineered nanomaterials is still in its infancy, there are significant knowledge gaps regarding the potential long-term health and environmental impacts of mass-produced nanoparticles used in novel technologies, such as biosensors [12].

Based on our literature review, which reveals a high degree of uncertainty regarding the potential negative impacts of wide dissemination of engineered nanomaterials in the environment, we suggest the adoption of precautionary frameworks such as the One Health approach as a necessary step toward the responsible scaled up production of nano biosensors and other nano-enabled technologies.

One Health is a transdisciplinary integrated approach that focuses on the interconnectedness among people, animals, plants, and their shared environment. The overall goal of this approach is achieving optimal human and environmental health outcomes [13,14]. A One Health approach to biosensor development could create new avenues and opportunities for interdisciplinary collaboration that bridges the gap between the fields of biosensor development (engineers, chemists, etc.), environmental toxicology (environmental toxicologists, environmental chemists, etc.), and human health (human toxicologists, public health practitioners, etc.). Herein we present several factors with an important role in toxicity (size, shape, concentration, surface chemistry, and transformations) in terms of materials commonly used in biosensors (nanometals, nanometal oxides, carbon-based nanomaterials, inorganic two-dimensional nanomaterials, and composite or alloy nanomaterials). We encourage the integration of a holistic consideration of nanomaterial toxicity in the development, scale-up, and disposal of biosensors that balances the benefits provided by the biosensor with the risk associated with uncontrolled nanoparticle release to the environment. We also identify the need for toxicology studies that bridge the current gaps in the literature to facilitate this effort.

## 2. Methods

The PRISMA framework was applied for structuring our literature review. Herein we present the steps used in identifying, selecting, and reviewing articles on the toxicity of nanoparticles. We also describe the components of the methodological framework including eligibility criteria, information sources, search strategy, and selection process as presented in the PRISMA 2020 checklist.

To achieve the objectives outlined in the introduction of this review, we performed searches of the Web of Science all databases (Web of Science Core Collection, Biosis (Citation Index & Previews), Current Contents, Data Citation Index, Derwent Innovations Index, KCI-Korean Journals, MEDLINE, Russian Science Citation Index, SciELO Citation Index, and Zoological Record). Website searches (Google) were also used to locate potential resources.

In preliminary literature searches, studies were included if they discussed the toxicity of nanoparticles utilized in biosensor development. Five categories of nanomaterials were identified to guide the focus of the review: (1) nanometals, (2) nanometal oxides, (3) carbon-based nanomaterials, (4) inorganic two-dimensional nanomaterials, and (5) composite or alloy nanomaterials. Secondary literature searches further guided the inclusion or exclusion of studies from the review and the subgrouping of studies for synthesis. Within each of the five categories of nanomaterials outlined above, papers were subdivided into specific metals (copper, gold, platinum, silver, and palladium), metal oxides (oxides of copper, iron, titanium, and zinc), carbon-based nanomaterials (graphene, graphene oxide, reduced graphene oxide, carbon quantum dots, and fullerenes or buckyballs), and inorganic two-dimensional nanomaterials (phosphorene, hexagonal boron nitride, and molybdenum disulfide) based on their reported use in biosensor development (Table 1). Category five, composite and alloyed nanomaterials, was not subdivided into individual combinations of nanomaterials because it was not practical to cover each subdivision. Instead, combinations of materials were discussed in the categories of (1) nanocomposites, defined here as heterogeneous materials comprised of at least one nanoscale (1 to 100 nm) component distributed on a substrate material, [15] and (2) nanoalloys defined here as heterogeneous materials consisting of two or more metal nanoparticles [16]. Keywords used to collect articles at this stage were: metal oxide *, silver, copper, “copper oxide *”, gold, platinum, palladium, “iron oxide *”, “titanium dioxide *”, “zinc oxide *”, graphene, “graphene oxide”, “carbon quantum dot *”, fullerene *, buckyball *, “reduced graphene oxide”, “multi-walled carbon nanotube *”, “single-walled carbon nanotube *”, “carbon nanotube *”, composite *, alloy *, phosphorene *, “hexagon * boron nitride *”, “molybdenum disulfide”, and “carbon nanoparticle *”. A total of 39,814 articles were received at this stage.

**Figure 1:**
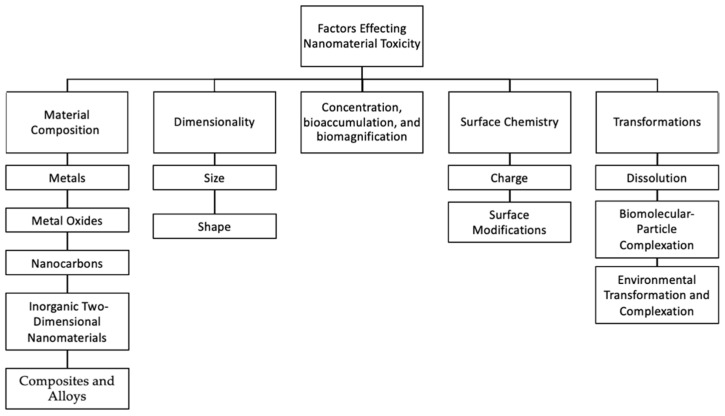
The key factors of nanomaterials affecting their toxicity: material composition, dimensionality, concentration bioaccumulation, biomagnification, surface chemistry, and transformations.

Upon reviewing trends in the collected literature five factors central to nanomaterial toxicity were identified to group literature in the review: (1) material composition, (2) dimensionality, (3) concentration, bioaccumulation, and biomagnification, (4) surface chemistry, and (5) transformations. These factors were further subdivided based on secondary in-depth reviews of the literature (Figure 1). Keywords applied at this stage included: size, shape, concentration, dissolution, “material composition,” dimensionality, bioaccumulation, biomagnification, “surface chemistry,” transformations, charge, capping, and functionalization.

The authors performed the preliminary collection of information independently; however, the secondary screening of studies was done collaboratively. Studies were first screened using search refining functions native to the web interface to decide whether a study met the inclusion criteria of the review. For example, due to the large number of articles returned in the initial search, we refined the search criteria to exclude document types, such as patents, abstracts, meetings, etc. from the results. We also prioritized results published within the last ten years (2012 to 2022). However, select studies outside of the 10-year range were included if they provided valuable fundamental knowledge. The search was further refined to exclude articles not written in English. A total of 19,021 results were received after applying these automatic exclusion criteria.

Given the large number of articles obtained, reviewers paired down the articles by searching within the results for a single material single factor combination. For example, the combination of gold and charge returns 355 results. These results were sorted by relevance, and roughly the top 100 titles and abstracts were screened. This process was repeated for each combination. Articles were removed if the abstract’s focus did not align with the review’s objectives or if the article did not present useful data on toxicity. For example, if the abstract or title did not include the mentioning of a methodological framework (model organisms, study length, independent and dependent variable) or endpoints (LC_50_ or EC_50_, estimates of the acute no-observed effect concentration (NOEC), behavioral observations, reproductive success, growth, survival (%), hatchability (%), microbial communities, toxicity mechanisms, genetic and epigenetic changes, gene expression, etc.) relevant to the domain of toxicity they were excluded. Studies included in the review were analyzed and summarized in tables, figures, or text.

Heterogeneity among results was explored to identify which factors (nanomaterial size, nanomaterial shape, model organism tested, etc.) differed among studies that could explain inconsistent results. Scientometric analysis was also preformed to map the current literature topics in the domain of nanotoxicity. A literature search on the Web of Science (ClarivateTM) limited to articles published in the past ten years, using the keywords “toxicity” AND “nano *” returned 56,828 articles. The number of articles published each year has increased steadily. In 2012 a total of 2333 articles were published while in 2021 a total of 8071 articles were published. These articles were most often published in the International Journal of Nano Medicine, RSC Advances, ACS Applied Materials Interfaces, Chemosphere, Science of the Total Environment, Scientific Reports, Journal of Hazardous Materials, Nanomaterials, Nanotoxicology, and nanoscale (top ten journals listed in order from most published in to least).

**Figure 2:**
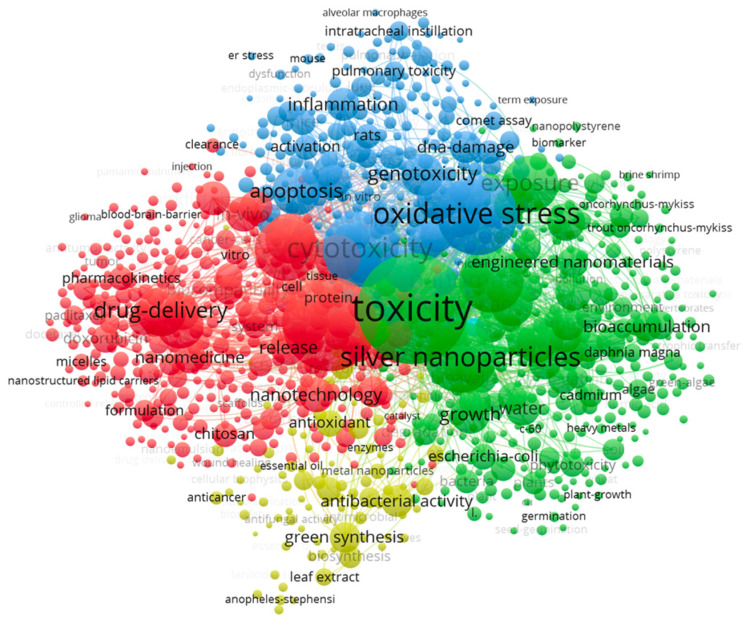
Network Visualization for 100 keywords from articles (English language; 2012 to 2022) retrieved with the keywords “toxicity” AND “nano *” on Web of Science all databases.

VOSveiwer was also used to visualize the co-occurrence of keywords from the top 10,000 (sorted by relevance) articles retrieved from the Web of Science search (full counting method) (Figure 2) [17]. Keyword occurrence was set to 10 and keywords were screened to remove redundancies (ex://drug delivery and drug-delivery). In VOSviewer the weight of occurrence relates to the size of the keyword label. In Figure 2, we see that terms such as toxicity, cytotoxicity, oxidative stress, drug-delivery, and silver nanoparticles have a high occurrence. VOSviewer also clusters keywords. In this case four clusters were formed. The red cluster centers around the theme of drug-delivery applications. The Blue cluster centers around the theme of cytotoxicity and the green cluster centers around the theme of toxicity. The yellow cluster is not well defined. In our analysis we generally focused on keywords from the toxicity (green) and cytotoxicity (blue) clusters.

**Figure 3:**
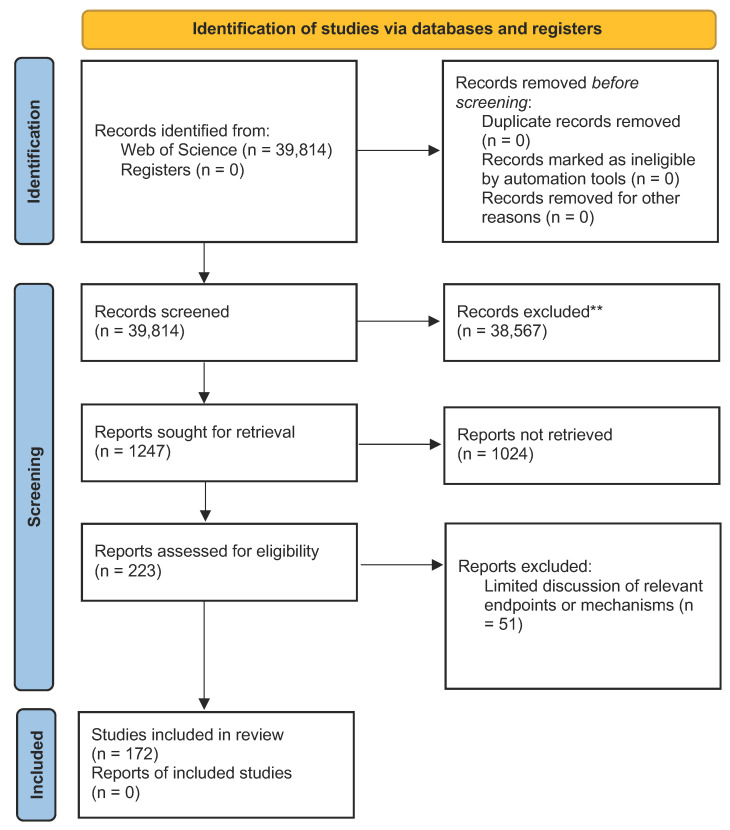
PRISMA 2020 flow diagram for new systematic reviews which included searches of databases and registers only covering the domain of nanotoxicity.

The data collection and selection process followed the logic presented in the PRISMA 2020 flow diagram (Figure 3) and resulted in the inclusion of 172 nanotoxicity articles. Within each article retrieved for review in the toxicity domain, data on a methodological framework and endpoints were collected.

## 3. Factors Affecting Toxicity

### 3.1. Material Composition 

Biosensors often pair biological recognition elements with metallic nanoparticles, metal oxide nanoparticles, nano polymers, and carbon-based nanomaterials [18,19,20,21,22]. These individual nanomaterials suitable for biosensor fabrication have associated toxicity scales dependent on the material composition. For example, ZnO, TiO_2_, SiO_2_, and Al_2_O_3_ of the same starting size (20 nm) demonstrate differing levels and mechanisms of in vitro toxicity in human fetal lung fibroblasts (HFL1) [23].

#### 3.1.1. Metals and Metal Oxides

Nanometals and nanometal oxides are often utilized for biosensor development because of their optical properties, electrochemical properties, ease of functionalization, cost-effectiveness, and biocompatibility (Table S1 in the Supplementary Materials). However, metals and nanometal oxides also have associated toxicity in different model organisms (Table 2). Furthermore, the toxicity associated with each metal or metal oxide differs based on their chemical compositions [23]. Here we present sample studies for nanoparticles commonly used in biosensors, including nanometals like copper, gold, platinum, silver, and palladium (Table 2) and nanometal oxides like copper oxide, iron oxide, titanium dioxide, and zinc oxide (Table 3). Herein we observe that the conclusions regarding toxicology and health impacts are not in total agreement. On a closer view of the methodologies used in the different studies, we could observe a lack of standardization in approaches, which explains the heterogeneity of toxicological outcomes. For example, several studies focus on the influence of only one or two variables (e.g., nanomaterial composition and dose) but fail to control for other important factors such as nanoparticle size, shape, and surface charge (presented here as zeta potential).

#### 3.1.2. Nanocarbons

Nanocarbons are often used in biosensing because of their high conductivity, high surface-to-volume ratio, and functionalization capacity. Typical forms of carbon used in biosensor development include graphene, graphene oxide (GO), reduced GO (rGO), carbon quantum dots, fullerenes (a.k.a., buckyballs) [63], and carbon nanotubes (CNTs). Our recent literature search on the Web of Science (ClarivateTM) limited to articles published in the past five years, using the keywords “carbon” AND “nano *” AND “biosensor,” returned 5622 articles with 300 to 550 articles published each year. In the past five years, numerous nanocarbon-based biosensors have been developed for the detection of various pathogens [64,65,66,67] and gasses [68,69,70,71], as well as in biomedical [72,73,74,75], food safety [76,77,78,79], and public and environmental health applications [80,81,82,83].

##### Carbon Nanoparticles

The toxicity of carbon nanoparticles such as onion-like carbon nanoparticles (OCNPs) derived from grilled *Scophthalmus maximus* (turbot) has been studied in mouse osteoblasts cells and zebrafish. OCNPs were able to enter cells easily but caused little damage other than the production of reactive oxygen species (ROS) (>20 mg/L). In zebrafish, exposure to OCNPs resulted in the production of ROS (2.5 mg/L), damaged lipids and proteins, and, at high concentrations, OCNPs caused mortality (maximum tolerance dose 25 mg/L and absolute lethal concentration 1000 mg/L). These findings suggest that prolonged exposure to OCNPs harms organisms [84].

##### Graphene

Graphene is a two-dimensional, one carbon atom thick, material with a hexagonal structure. While graphene and its derivatives are generally considered biocompatible, and it is currently one of the most popular nanomaterials for biosensors development, various studies have shown the toxicity of graphene and its derivatives (GO, rGO, etc.). The proposed methods of toxicity reported include physical or mechanical damage, ROS production, apoptosis, inflammation, autophagy, necrosis, DNA damage, and mitochondria damage in an interconnected manner [85]). For example, in a study of pristine graphene (pG) toxicity in kidney cells, pG was shown to induce DNA fragmentation (>50 μg/mL) [86].

Several studies demonstrate the toxicity of graphene to marine organisms. For example, in a study of zebrafish embryos, pG-induced embryonic mortality (>30 μg/mL), delayed hatching (25 μg/L), morphological defects, as well as bradycardia and tachycardia (10 and 25 μg/L) [87]. In zebrafish (*Danio rerio*) graphene has been shown to induce pathological tissue damage and activation of the antioxidant system in the gills, muscles, and brain, with the brain being the main organ affected (5 mg/L and 50 mg/L) [88].

##### Graphene Oxide

Graphene oxide (GO) is a graphene derivative with oxygenated functional groups [89]. In marine ecotoxicity studies, GO has displayed toxic effects in organisms across the trophic chain. For instance, in the marine bivalve *Crassostrea virginica*, GO induces acute and chronic effects from both short- and long-term exposures. In short-term exposures of 1 to 10 mg/L (72 h), GO caused adverse health effects via epithelial inflammation and oxidative stress [90]. In long-term exposures of 2.5 and 5 mg/L (14 days), GO also induced oxidative stress, as well as elevated glutathione-s-transferase (GST) enzyme levels in gill and digestive tissues, leading to adverse cellular damage [91]. In zebrafish, GO increased early-stage apoptotic and necrotic cells in gill tissue (2 mg/L and 20 mg/L). GO was also linked to ROS generation (10 and 20 mg/L) and cellular injury (2 mg/L, 10 mg/L, and 20 mg/L) in the gills. In liver cells, GO led to changes in cellular morphology and untimely cell rupture and necrosis [92]. In the brine shrimp (*Artemia salina*) GO affected both the cyst and larval stages. Concentrations of 400 and 600 mg/L reduced the hatchability of capsulated and decapsulated cysts after 36 h of exposure. In the larval stages, GO increased mortality and decreased individual larvae’ swimming speed, body lengths, and body weights [93].

In studies of unicellular organisms (*Saccharomyces cerevisiae, Candida albicans, and Komagataella pastoris*) and prokaryotes (*Pseudomonas fluorescens*) GO significantly inhibits growth by binding iron and thereby inhibiting iron-related physiological and metabolic processes (10 mg/L to 160 mg/L) [94]). In vivo studies of *Acheta domesticus* demonstrate that short-term exposure (10 days) to 200 μg/g GO induced oxidative stress, DNA damage, and degenerative damage in cells of the gut and testis [95]. Interestingly, in rat corneal epithelial cells (rCECs), repeated short-term GO exposure also displayed optical toxicity via intraocular inflammation, apoptosis in the cornea, cytotoxicity in the corneal epithelial cells, and iris neovascularization [96].

##### Reduced Graphene Oxide

Reduced GO (rGO) is yet another derivative of graphene derived from the chemical, thermal, electrochemical, and photochemical reduction of graphene oxide [97]. In a study of zebrafish embryos, rGO inhibited hatching success (5 mg/L = 75.9%, 1 mg/L = 71.4%, 50 mg/L = 28.1%, and 100 mg/L = 25.8%) and reduced the length of larvae (10 mg/L to 100 mg/L) [98]. In a study of mouse dams, rGO also displayed negative reproductive effects. In the late gestational stage, high doses (25 mg/kg) of rGO killed most mice damns while at low (6.25 mg/kg) to moderate (12.5 mg/kg) doses of rGO caused abortion in all mice. In early gestational stages, rGO did not preclude the delivery of pups; however, it did cause fetal malformations [99].

Various studies on the toxicity of rGO show a time dependency. For example, in bacterial biofilms (*Escherichia coli* and *Staphylococcus aureus*), rGO hindered growth and film formation (≥50 mg/L); however, the effects diminished after 24 h and stopped after 48 h. Similarly, rGO increased ROS in the early stages, but levels returned to normal in the medium and late stages [100]. Time-dependent toxicity was also witnessed in zebrafish liver cell lines (24 to 72 h). However, in this case, rGO induced ROS production at higher concentrations (25 and 100 µg/mL) after 72 h. Interestingly, in this case, rGO was not found in the cells suggesting that it induced toxicity through interactions with the cell membrane [101]. Finally, in PC12 (neural cell lines), rGO has been shown to induce time-dependent toxicity via apoptosis (20 µg/mL, 40 µg/mL, and 60 µg/mL) and cell cycle arrest (20 and 50 µg/mL) [102].

Carbon quantum dots (CQDs) are zero-dimensional carbon-based nanomaterials [103]. CQDs are generally considered biocompatible [104]. However, this is not always the case. In a study of zebrafish (*Danio rerio*), zooplankton *(Daphnia magna*), and phytoplankton (*Scenedesmus obliquus*), CQDs showed negligible toxicity in *Danio rerio* but exhibited considerable acute toxicity in *Daphnia magna* and *Scenedesmus obliquus* [105].

##### Fullerenes

Fullerenes (C_60_), also known as buckyballs, are an allotrope of carbon. In studies of the toxicity of C_60_ emulsions in mice, C_60_ negatively affected lung and sperm cells. In the lungs, C_60_ induced morpho-functional and inflammatory hazards. Exposure to C_60_ also damaged sperm cells and increased the risk of infertility [106]. Studies of *Daphnia magna* and zebrafish show that chronic C_60_ exposure induced behavioral and physical abnormalities at concentrations at and over 5 mg/L and 2 mg/L, respectively [107,108]. These findings are important to understanding the toxicity of C_60_ in aquatic ecosystems.

##### Carbon Nanotubes

Carbon nanotubes are hollow nanosized cylindrical tubes composed of graphite sheets (graphene). Nanotubes are classified as either single- or multi-walled depending on the number of layers [109,110]. Studies of single- and multi-walled carbon nanotube (SWCNTs and MWCNTs, respectively) toxicity have shown deleterious effects in cells, mammals, aquatic organisms, and plants.

In seeds of *Cucurbita pepo* L., MWCNTs impaired morpho-physiological and biochemical processes leading to decreased germination, root and shoot length, and biomass accumulation [111]. In *Hyoscyamus niger* seeds, SWCNTs have shown concentration-dependent toxicity. At high concentrations (1000 μg/mL), SWCNTs inhibited seedling germination and performance, increased cellular injury indices, and altered antioxidant enzyme activities. However, when SWCNTs were applied at low concentrations (125 and 250 μg/mL), seeds in drought-like conditions saw reduced drought stress [111].

MWCNTs have also been shown to affect health and reproduction in the model aquatic animal *Xenopus tropicalis*. Specifically, MWCNTs inhibited the growth of the body (testis, ovaries, and fat), accumulated in the lungs, and decreased the rates of fertilization and survival for embryos [112]. In common carp (*Cyprinus carpio*), SWCNTs altered brain and testicular function by downregulating essential steroidogenic and transcription factor genes. SWCNTs exposure altered the structure of the testis, decreased cell viability in culture, increased reactive oxygen species levels, led to apoptosis, and caused DNA damage [113].

Research on SWCNTs toxicity in cells (normal human astrocytes) shows that SWCNTs have a disruptive effect on immune response via the down- and up-regulation of a subset of genes [114]. In studies of rats, SWCNTs inhibited mitochondrial function and induced hepatotoxicity [115]. In mice, it has been suggested that the uptake of SWCNTs by macrophages activates transcription factors that lead to oxidative stress, inflammation, and severe pulmonary granuloma formation (0.5 mg) [116]. It is important to note that SWCNTs and MWCNTs display pulmonary toxicity [117].

Several cellular studies have found that MWCNTs promote the accumulation of lipids via the induction of endoplasmic reticulum (ER) stress [118,119,120]. The accumulation of lipids in cells, such as aortic smooth muscle cells, can have adverse long-term health effects by contributing to conditions such as atherosclerosis [120]. MWCNTs have also been shown to affect lipid metabolism in a transgenerational animal study. Mice exposed to MWCNTs produced offspring with decreased weight and histopathological changes in liver tissue. Specifically, MWCNTs affect genes that mediate fatty acid synthesis in the liver, thereby disrupting liver function and leading to the accumulation of lipid droplets in hepatocytes [121]. Interestingly, MWCNTs also displayed transgenerational toxicity in nematodes. In this case, via the activation of germline long non-coding RNA linc-7 [122].

#### 3.1.3. Inorganic Two-Dimensional Nanomaterials

Emergent inorganic 2-D nanomaterials with atomically thin sheet morphology are characterized by unique structural and physicochemical properties. These materials demonstrate significant potential for widespread environmental and health applications, including biosensing. Unlike graphene, which has a zero bandgap, non-carbon 2-D nanomaterials possess a tunable bandgap, allowing modulation of their electronic, catalytic, optical, and photocatalytic properties. The family of emergent 2-D non-carbon nanomaterials is very diverse and includes materials that can be distinguished from each other by their structure and properties affecting their stability, behavior in the environment, bioavailability, and toxicity. For example, non-metallic phosphorene is exfoliated from black phosphorus, has a puckered structure, bandgap between 0.3 to 2.3 eV, and absorbance in the IR and visible light spectrum, while metalloid hexagonal boron nitride (h-BN) has a honeycomb structure with a wider bandgap of 4–6 eV and absorbance in the UV spectrum [123]. Phosphorene is more soluble, while h-BN is more stable in aqueous media. There are also representatives from transition metal dichalcogenides, e.g., Molybdenum disulfide (MoS_2_) with a triatomic sandwich-like structure between two layers of sulfur [124].

Among the properties influencing the toxicities of 2-D inorganic materials are the number of layers, surface size, shape, and area [125]. Methods used for exfoliation and surface modification can also affect the toxicity of these 2-D materials [126]. Exfoliation can mitigate toxicity, as has been shown in a study of in-vitro toxicity for exfoliated versus aggregated MoS_2_ [127]. Corazzari et al. (2014) detected an increase in ROS in human tumor lung cells after exposure to MoS_2_ but only at the highest concentrations analyzed [128]. Modification of MoS_2_ surface with chitosan improved therapeutic effect in cancer cell lines compared to non-functionalized MoS_2_ [129]. Among the non-carbon 2-D nanomaterials, black phosphorus was found to be more toxic than metal dichalcogenides [130]. However, some of the dichalcogenides also contain tellurium (Te) or selenium (Se), which can be released from these materials in different environments and induce human and animal toxicity [126]. The toxicity of black phosphorus to bacteria and human cells, as well as, mice has been demonstrated previously due to induced oxidative stress and impairment in membrane integrity [131]. When mice were exposed to black phosphorus quantum dots, the animals showed signs of oxidative stress but recovered [131]. The concentration-dependent toxicity was observed for *C. elegans* mortality and reproduction after exposure to phosphorene, with reproductive toxicity being a much more sensitive endpoint showing inhibition at 2 mg/L [132]. Phosphorene is stable in water only in anaerobic conditions while it oxidizes in the presence of oxygen [133], and its stability, interactions with biological surfaces, and, thus, toxicity can be affected by the degree of oxidation. There are still many unknowns for behavior and toxicity of the 2-D inorganic nanomaterials, especially under different environmental and in-vivo conditions, that need to be examined further before these materials are utilized even more broadly.

#### 3.1.4. Composites and Alloys

Nanocomposites and nanoalloys display unique and synergistic effects, which make them useful in biosensing [134,135,136]. Nanocomposites can be generally classified as (1) a macroscale matrix with a nanoscale filler (matrices with nanoparticle fillers, nanofiber fillers, laminar fillers, and aerogels) or (2) a nanoscale matrix with a nanoscale filler (core-shell and multi core-shell nanoparticles, coated and multi-coated nanoparticles, decorated and multiphase decorated nanoparticles, multiphase clusters, encapsulated phases with active cores, mixed nanoparticles, barcode nanoparticles, nanoparticle liposomes, and hollow nano shells within nano shells) [137].

While the toxicities of single nanomaterials have been studied in various organismal studies, uncertainties arise concerning the unknown effects of exposing an organism to lethal and sublethal doses of nanomaterial composites. In many cases it is not well known how the interaction of composite materials will affect toxicity since combined effects from different nanomaterials could be additive, synergistic, or antagonistic. For example, in the model organism *C. elegans* studies have shown silver nanoparticles cause mortality, growth inhibition, reproductive toxicity, and oxidative stress [39,138]. Similarly studies have shown that graphene oxide effects lifespan and causes oxidative stress, neurotoxicity, and reproductive toxicity [139,140]. However, studies on the combined effects of silver and graphene oxide in *C*. *elegans* are lacking. Unfortunately, this is the case with many other organisms as the literature on the toxicity of nanocomposites is considerably limited in comparison to the literature on single/pristine nanomaterials. For example, while our literature search on the Web of Science (ClarivateTM) using the keywords “toxicity” AND “nano *” returned 56,828 articles, a search of the keywords “toxicity” AND “nano *” AND “composite *” returned only 4277 articles.

**Figure 4:**
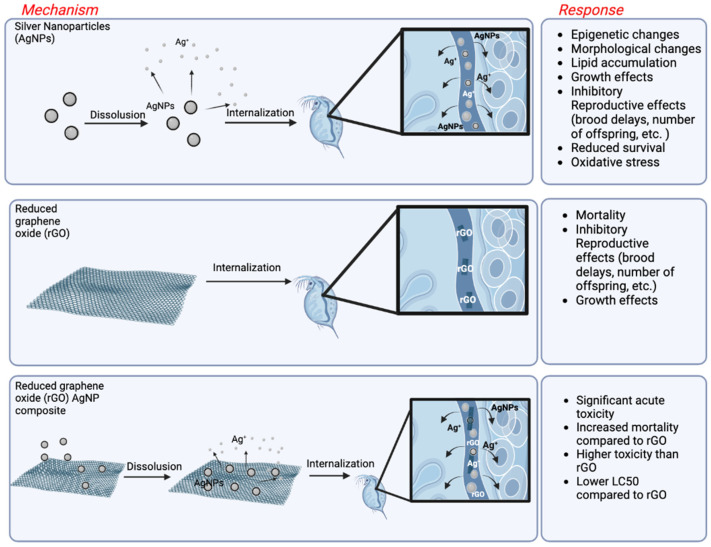
Schematic representation of the individual and composite toxicity [142] of silver nanoparticles [39,138] and reduced graphene oxide nanosheets [142] on the organism Daphnia magna. “Created with BioRender.com (accessed on 18 November 2022).”

In one nanocomposite toxicity study, Yin et al. (2020) examined the effects of six carbon-based nanocomposites (GO-Au, GO-Ag, GO-Pd, GO-Fe_3_O_4_, GO-Co_3_O_4_, and GO-SnO_2_) on two algae species (*Scenedesmus obliquus* and *Chlamydomonas reinhardtii*). Among the endpoints studied were algal growth, cell permeability, and oxidative stress. The difference in toxicity among tested nanocomposites was associated with the specific metals in the nanocomposites, with GO-Ag causing the strongest growth inhibition, followed by GO-Pd and GO-Co_3_O_4_. The cells exposed to GO-Au and GO-Fe_3_O_4_ also demonstrated growth inhibition, but the impact was less severe and did not differ from the toxicity observed after GO exposure alone. The increase in oxidative stress leading to cell membrane damage occurred in response to either GO-Ag, GO-Au, or GO-Pd exposures, and the observed damage was significantly higher compared to GO exposure alone. Association with Ag had the strongest adverse impact on membrane damage. The observed toxicity was species-specific and could have been associated with stronger metal adsorption to more hydrophobic cell surfaces of *C. reinhartii* compared to *S. obliquus.* One of the toxicity mechanisms observed was the release of metal ions and their adsorption and interaction with the cell surfaces resulting in oxidative damage. Interestingly, the surface area differed among nanocomposites with GO-Ag having the largest surface area [141]. A similar trend was observed with in the model organism *Daphnia magna* (Figure 4) where rGO-Ag nanocomposites demonstrated higher mortality than pristine rGO sheets. In this case there was a considerable release of silver ions from the silver nanoparticles in the composite. These findings suggest that at the same exposure doses, conjugated graphene-silver nanoparticle trigger increased toxicity on *D*. *magna* compared to either material alone [142].

Another study assessed the antibacterial effects of silver nanowires and silver nanowire-carbon nanotube composites using *E. coli*, *S. aureus*, *Methicillin-resistant Staphylococcus aureus* (MRSA), and *S. saprophyticus* as model organisms. The materials tested included silver nanowires, silver nanowire-carbon nanotube composites, silver nanowire-carbon nanotube mixtures, silver ions, and carbon nanotubes. All materials studied stopped cell growth except for carbon nanotubes which showed no antibacterial activity. Silver ions were the most efficient in inhibiting growth. In contrast, silver nanowires, silver nanowire-carbon nanotube composites, and silver nanowire-carbon nanotube mixtures demonstrated minimum inhibitory and bactericidal concentrations that were not significantly different from each other. When considering bacterial growth kinetics, carbon nanotubes showed no effect. In contrast, silver nanowires and silver nanowire-carbon nanotube composites showed effects in the lag phase and exponential growth time prior to reaching the stationary phase. Interestingly, *E*. *coli* and *S. aureus* were more sensitive to silver nanowire-carbon nanotube composites. Given their findings, the authors concluded that carbon nanotubes did not affect the antibacterial effects of silver nanowires [143]. It is important to note that there is an observed increase in toxicity between pristine carbon nanotubes and the composites and mixtures, most likely linked to the presence of silver nanoparticles.

The toxicity of nanocomposites was further investigated in a study of the effects of copper-carbon nanotube complexes and iron-carbon nanotube complexes on bacterial bioluminescence and seed germination in *Escherichia coli* and *Lactuca sativa* L., respectively. The results of this study show that the mean toxicity of copper-carbon nanotube complexes increased with increasing carbon nanotube content, while the mean toxicity of iron-carbon nanotube complexes decreased with carbon nanotube content in both organisms. The harmful effects of carbon nanotubes were limited. Therefore, the exact cause of this differential toxicity was unclear. Possible explanations proposed by the authors include the differences in individual metal toxicity (copper demonstrated much higher toxicity), carbon solubility, and chemical transformations between the metals [144].

Nanoalloys, as mentioned above, are heterogeneous materials consisting of two or more metal nanoparticles [16]. Studies of nanoalloy toxicity highlight the importance of dissolution and material composition (Table 4). For example, Hahn et al. (2012) carried out a study comparing the toxicity of cobalt, nickel, and titanium nanoparticles, and nickel-iron, and nickel-titanium alloys in human coronary artery endothelial cells (hCAEC) and human coronary artery smooth muscle cells (hCASMC). They found that cobalt and nickel nanoparticles displayed the highest toxicity, followed by nickel-iron and nickel-titanium alloys, and finally, titanium nanoparticles (Ni, Co > NiFe, NiTi > Ti). From these results, they concluded that observed toxicity was related, in part, to the release of ions. Therefore, because the absolute amount of nickel ions is lower in the alloys, the cytotoxic concentration threshold of alloy nanoparticles was one magnitude higher than the pure nanoparticles [145]. Similar results were demonstrated in a study of silver–gold alloy nanoparticles (AgAu, 68.7 wt% Ag and AgAu, 35.4 wt% Ag) in gram-positive bacterium *S. aureus* and Human gingival fibroblasts (HGFib). In this case, the antibacterial and cytotoxic effects of silver nanoparticles, which are known to dissolve, were decreased when gold was alloyed [146]. The effects of dissolution on toxicity will be further investigated later in this review.

### 3.2. Dimensionality

**Figure 5:**
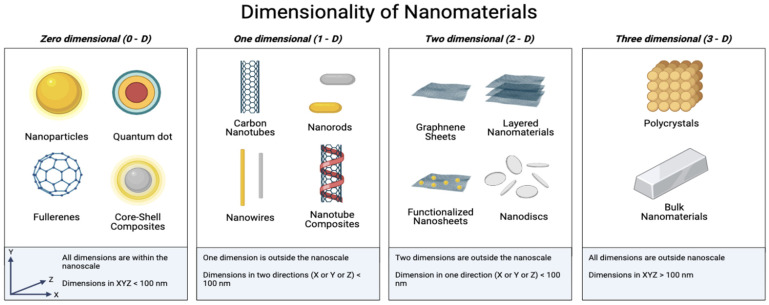
Nanomaterials are broadly classified as zero-dimensional (0-D), one-dimensional (1-D), two-dimensional (2-D), or three-dimensional (3-D) based on the number of dimensions measuring within the nanoscale. Adapted from “Classes of Nanoparticles”, by BioRender.com (accessed on 2 November 2022). Retrieved from https://app.biorender.com/biorender-templates.

Materials can be classified as nanomaterials if at least one-dimension measures less than 100 nm. Nanomaterials can be classified as zero-dimensional (0-D), one-dimensional (1-D), two-dimensional (2-D), or three-dimensional (3-D) (Figure 5).

Nanomaterials of differing dimensions are utilized and combined into nanocomposite materials in biosensors [7,152,153,154,155,156,157], and thus, a deeper understanding of the effects of dimensionality via systematic study is needed. Toxicity associated with dimensionality has been discussed by reviews on individual 0-D [158], 1-D [158], 2-D [126,159], and 3-D materials [160,161]. However, it is difficult to determine the direct effect of dimensionality on toxicity. For example, Castro Cardoso da Rosa et al. (2021) reviewed the effect of dimensionality on the toxicity of carbon nanostructures in model organisms by examining the toxicity of 0-D fullerenes, 1-D carbon nanotubes, 2-D graphene derivatives (graphene, graphene oxide, reduced graphene oxide, and graphene metallic nanoparticle nanocomposites), and 3-D graphene derivatives (graphene foam, graphene oxide foam, graphene nano-conches, 3-D graphene, 3-D graphene sheets, and, graphene hydrogels). They determined that it was not possible to infer a direct relationship between dimensionality in 0-D, 1-D, and 2-D materials. However, they concluded that 3-D materials demonstrate lower toxicity, most likely due to their more stable and compact structures, which make them less bioavailable and biodegradable [162].

Understanding of deferential toxicity related to dimensionality is complicated by the many confounding factors caused by a lack of consistency in experimental conditions (model organisms, length of exposure, etc.) and material preparations (functionalization, capping, etc.), which may play an important role on the observed toxicological outcomes (Table S2 in the Supplementary Materials). For example, within this review, the toxicity of 0-D nanomaterials can be explored by examining the toxicities of metallic nanoparticles, fullerenes, and carbon quantum dots. We can determine that metallic nanoparticles, fullerenes, and carbon quantum dots all have the potential to generate oxidative stress in cells, but contributions from factors such as shape, materials composition, concentration, etc., must also be considered before attributing toxicity to dimensionality. As such, multicriteria studies directly comparing the toxicity of nanomaterials within a single-dimensional classification are needed. To further examine potential toxicity related to dimensionality, we discuss the influence of material size and shape.

#### 3.2.1. Size

**Figure 6:**
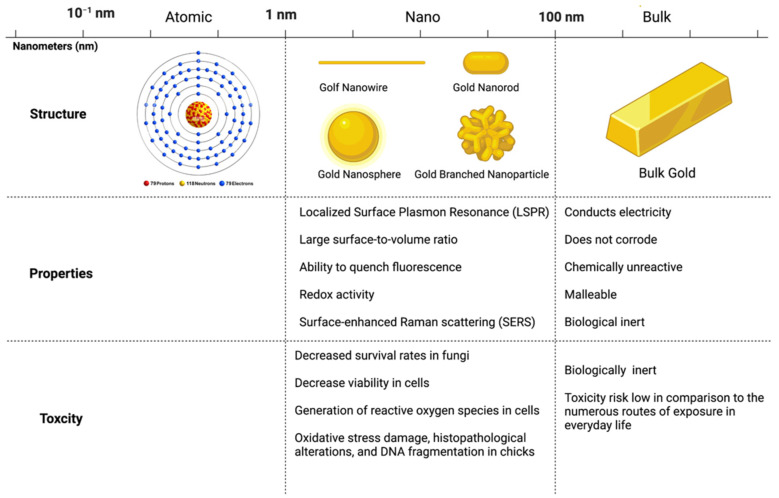
Schematic Representation of the differences between bulk and nanoscale gold. At the nanoscale, gold has interesting properties distinct from the bulk form [168,169] and displays toxicity not seen in bulk gold [30,32,34,165,170]. Adapted from “Size Comparison (Layout)”, by BioRender.com (accessed on 2 November 2022). Retrieved from https://app.biorender.com/biorender-templates.

Nanoparticle size contributes to many behaviors that are distinct from the material behaviors of the bulk form. For example, bulk metals often have a shiny appearance caused by freely roaming electrons. At the nanoscale, however, surface plasma resonance begins to dominate the optical properties of the metal nanoparticles creating colors distinct from those observed in the bulk material. By altering the size and shape of a nanoparticle, it is possible to tune the surface plasmon resonance [163]; therefore, numerous biosensors utilize this mechanism in optical detection platforms [164] (Figure 6). While size-dependent properties show promise in biosensing applications, they also create unique mechanisms of toxicity. For example, the bulk form of gold is considered biologically inert [165]. In contrast, gold nanoparticles have been shown to cause in vitro and in vivo toxicity in cell lines and model organisms (Table 2) (Figure 6). Numerous studies show nanoparticles are more toxic to environmental and human health than their bulk counterparts [58,166,167].

The factors that contribute to size-dependent changes in chemical reactivity and material properties between bulk and nanomaterials can be explained through several interrelated mechanisms. For example, when nanoparticle size decreases, the proportion of atoms at or near the surface increases significantly. As a result, the surface becomes more reactive. Likewise, when the size decreases, defects on and near the surface appear in the form of changes in vacancies, bond length, and bond angle [171]. Due to their size and, consequently, their high specific surface area, nanoparticles can penetrate organisms down to the cellular level, accumulate in tissues and organs, and interact with cellular components [172]. Studies of aquatic organisms show nanoparticles accumulate in fish tissues (liver, intestine, gill, and muscles), urchin embryos, and algae [173,174,175,176,177]. Similarly, in human, animal, and cell line studies, nanoparticles were shown to accumulate in the cells of the brain, kidneys, liver, spleen, heart, and lungs [178,179,180,181]. Once nanoparticles accumulate in cells, they can cause apoptosis, histological changes, necrosis, lysis, atrophy, and DNA damage

Within the nanoscale, researchers have also identified differential size-dependent toxicity of particles. Generally, toxicity is inversely proportional to size; however, the literature is inconclusive (Table S3 in the Supplementary Materials). For example, Tang et al. (2018) studied the short-term and acute (LD50) toxicity of copper nanoparticles (30 nm, 50 nm, and 80 nm) in male Sprague Dawley rats. They found that the LD50 of 30 nm particles was the lowest suggesting that 30 nm particles have higher toxicity in acute exposures. However, they concluded that 80 nm particles were the most toxic in short-term oral exposures [182]. These discrepancies in the literature can partly be attributed to several factors:

- Biodistribution of particles: nanoparticles have been shown to target and accumulate in different tissues and organs based on size [32,182,183].
- Uptake and excretion of particles: the uptake and excretion of particles have been connected to their size [183,184].
- Confounding factors: factors such as exposure time, administration routes, shape, model organism, etc., often vary across studies (Table S3 in the Supplementary Materials).

#### 3.2.2. Shape

Nanoparticles come in various shapes including tubes, rods, spheres, cubes, wires, triangles, ellipsoids, etc. which ultimately influence toxicity [185,186]. For example, in the soil organism *C. elegans*, silver nanoplates and irregular spheres inhibited growth and reproduction, while silver nanowires had a negligible effect [187]. Similarly, in the aquatic organism *Danio rerio*, silver nanoplates displayed higher toxicity than spherical silver nanoparticles. The nanoplates’ sharp and irregular surfaces likely contributed to their greater toxicity in both of these studies [188]. One theory to support this claim suggests that silver ions can more easily dissolve from the sharp edges. Thus, the toxic effect can be attributed to the ionic gradient instead of the nanoparticle itself. However, studies comparing the toxicity of silver nanoparticles and silver ions somewhat contradict this theory because they find that silver nanoparticles induce greater toxicity than silver ions. Alternatively, some researchers suggest that silver nanoplates have more reactive atoms on the surface and induce toxicity via direct contact combined with the dissolution of silver ions [188].

The literature proposes several factors that influence shape-dependent toxicity; however, the exact mechanisms are unclear, and more research is needed to fill this knowledge gap. For example, several reviews suggest that shape-dependent toxicity is closely linked to the uptake mechanism. Specifically, they presume that spherical nanoparticles are more likely to enter cells through endocytosis than nanotubes and fibers [189,190]. However, in a recent study of titanium dioxide nanoparticles, spherical fine particles, nanosquares, and nanotubes all entered cells via the formation of endosomes [191]. Alternatively, nanorods, wires, and fibers may display differential toxicity because of their length. For example, nanowires longer than the typical food source of the model organism being tested cannot be taken up [187]. Similarly, the contact angle of rod-shaped nanoparticles affects the rate of internalization. If the particle aligns with its short axis parallel to the cell membrane, it will be internalized faster than a particle with its long axis parallel to the cell membrane, while the rate of internalization of spherical nanoparticles is independent due to their symmetry [192].

Once internalized, the shape of a nanoparticle can affect biodistribution, accumulation, and excretion. In mice, spherical and star-like nanoparticles demonstrated a similar ability to penetrate the liver and spleen, while rods were less likely to penetrate and were excreted quickly. Star-like nanoparticles were uniquely able to enter the lungs [193]. This finding is interesting because another study found that gold nanostars exhibited the highest cytotoxicity compared to nanospheres and nanorods [194].

Nanocrystal morphology may also play a role in toxicity. For example, Chang et al. (2017) examined the effect of palladium nanocrystal morphology on in vitro toxicity profiles and in vivo oxidative injuries. According to the study, the enlargement of {100} crystallographic facets results in lower toxicity due to the higher material stability, which diminishes the post-internalization release of Pd(II) ions into the cell [195]. However, the authors mention that enlarging the {100} crystallographic facets may decrease the electrocatalytic activity of nano palladium crystals. In other words, making this material safer may also mean making it less efficient for electrochemical sensing applications [196].

### 3.3. Concentration, Bioaccumulation, and Biomagnification

Comparative studies of exposure to lethal and sublethal doses of nanoscale versus bulk, micro, and molecular scale materials are important for unveiling the differential mechanisms driving nanoparticle toxicity [197]. A study by Abdel-Khalek et al. (2015) compared the LC_50_/96 h of the aquatic organism *Oreochromis niloticus*, exposed to both bulk- and nano-copper oxide particles. In this case, the LC_50_/96 h of bulk copper oxide was 2205 mg/L; by contrast, only 150 mg/L of nano copper oxide was required to achieve 50% population death [198]. In other words, the lethality of the nanoparticles was significantly higher than the toxicity of the bulk material. When examining sublethal levels, the authors found that both nano and bulk particles induced biochemical alterations and oxidative stress in the liver and gill tissues of the studied fish. However, in most instances, copper oxide nanoparticles caused more toxic effects than bulk copper oxide, except at concentration thresholds where nanoparticles can rapidly form aggregates in suspensions. Similar studies have reached analogous conclusions [55,93].

Nevertheless, it is important to recognize that the available literature shows no generalizable correlations between the concentration regime of nanoparticles and their toxic effects on a given organism. In some cases, even relatively small variations in dosage can result in vastly unexpected toxicological outcomes. For example, Hassanen et al. (2020) assessed toxic effects in Cobb chicks from daily exposure to 5 ppm and 15 ppm of gold nanoparticles via drinking water ingestion. Experimental data included oxidant/antioxidant parameters, histopathological organizations, proinflammatory cytokine levels, and DNA assay. Surprisingly, the low concentration level (5 ppm) resulted in positive health outcomes with respect to the controls, such as increased growth performance and immune defense without affecting the histological structures of the internal organs. On the other hand, a relatively small increase in gold nanoparticle concentration (15 ppm) led to extensive cytotoxicity and genotoxicity in the animals [34]. Another study has also reported positive outcomes in soil microorganisms exposed to low doses of carbon nanotubes [199]. However, other model organisms have shown negative effects from exposure to similar doses of carbon nanotubes [111,113,114,116,117,118,119,120].

Another important consideration when analyzing the toxicological impacts of nanomaterials on human health and the environment is their capacity to bioaccumulate and biomagnify across the trophic chain. Judy et al. (2011) evaluated the plant uptake and the potential for trophic transfer from exposure to gold nanoparticles in soil using the model organisms *Nicotiana tabacum* L. *cv Xanthi and Manduca sexta.* The study showed compelling evidence of trophic transfer of nanogold from a terrestrial primary producer (*Nicotiana tabacum* L. *cv Xanthi)* to a primary consumer (*Manduca sexta*), as well as evidence of biomagnification in a terrestrial food web [200]. A similar study by Unrine et al. (2012) looked at the trophic transfer of gold nanoparticles along a simulated terrestrial food web. In this case, a soil organism *Eisenia fetida* (earthworms), was exposed to gold nanoparticles in artificial soil media and subsequently fed to juvenile *Rana catesbeina* (bullfrogs). The study concluded that gold nanoparticles dispersed in the soil might ultimately be transferred to higher-order consumers through detrital-based food chains [201]. When assessing biomagnification potential, the biology, physiology, and life history traits of the organisms involved at each trophic level are probable interplaying factors. Therefore, even for nanoparticles with similar physicochemical properties, prevailing community composition and local ecological conditions will significantly influence the nature and extent of trophic transfer. Overall, these studies stress the urgency of including dietary uptake as an exposure pathway and raise concern for the potential implications to a variety of eco receptors (including humans) from the widespread dispersion of engineered nanomaterials in environmental matrices.

### 3.4. Surface Chemistry

#### 3.4.1. Charge

The charge of a nanomaterial influences its biological interactions. For example, it is generally accepted that positively charged nanomaterials display a higher level of cellular interaction than negatively charged nanomaterials because of their capacity to interact with negatively charged cell membranes leading to cellular uptake or membrane damage [202]. In many studies, the surface charge is described through zeta potential measurement. In a study of morphology and surface charge-dependent cellular uptake of nanoparticles in phagocytic THP-1 macrophages and non-phagocytic A549 cells, a positive association between the zeta potential and cellular uptake was identified. In THP-1 macrophages exposed to polystyrene nanoparticles at a concentration of 50 μg/mL, three of four positively charged nanoparticles showed 15 to 21 percent cellular uptake. In contrast, two out of three negatively charged nanoparticles showed approximately 5 to 8 percent cellular uptake. This occurred, to a lesser extent, in non-phagocytic A549 cells [203].

This trend has also been observed in studies of iron oxide nanoparticles [204,205]. Positively charged iron oxide nanoparticles tend to interact with negatively charged membranes leading to considerable uptake. The significant and deep uptake of iron oxide nanoparticles, in turn, leads to greater toxicity of positively charged nanoparticles compared to negatively charged ones [205]. For example, in a study of superparamagnetic iron oxide nanoparticles, cell viability was lowered in all cell lines studied (HCM (heart), BE-2-C (brain), and 293T (kidney)) by positively charged particles [205]. However, it is important to note that this is not always the case. For example, negatively charged particles were the most toxic in a study of silver nanoparticle toxicity in human tumoral cell lines (U-937 and HL-60) [206].

It has been suggested that charged nanoparticles, whether positive or negative, are taken up more efficiently than their neutral counterparts. In a study of the surface charge and size-dependent uptake of graphene sheets by MCF7 cells, neutrally charged nanoparticles showed negligible uptake. In contrast, charged nanoparticles showed size-independent (positive) and dependent (negative) uptake efficacy. Positively charged particles entered via phagocytosis and clathrin-mediated endocytosis, while negatively charged particles entered via phagocytosis and sulfate-receptor-mediated endocytosis. Interestingly, positively charged graphene sheets always displayed higher toxicity irrespective of size [207].

Charge density has also been identified as an important determinant in the fate and toxicity of nanomaterials. In a study of carbon nanotubes, charge density was found to be a more accurate predictor of toxicity than zeta potential. When comparing five positively charged carbon dots of similar zeta potentials (+ 20.6 to + 26.9 mV) in vitro (THP-1, Calu3, and A549 cells) and in vivo (male Balb/c mice), only the particles with the highest charge densities (2.95 and 4.39 µmol/g) induced high cell viability loss, oxidative stress, mitochondrial dysfunction, and loss of lysosomal integrity in vitro and airway inflammation in vivo. These findings are interesting in that they suggest that the number of positive charges on the nanoparticle may be a better predictor of toxicity than the magnitude of the charge [208]. Likewise, charge density was identified as an important factor in a study of the adsorption of humic acid molecules onto gold nanoparticles. Positively charged nanoparticles with a low charge density showed less humic acid adsorption than high charge density nanoparticles. These findings help explain the mitigation of the toxicity of positively charged hydrophobic gold nanoparticles in *E. coli* by humic acid adsorption [209].

#### 3.4.2. Surface Modifications 

Biosensors are often functionalized to enhance their performance (sensitivity, selectivity, response time, etc.) [210,211,212,213] and biocompatibility [214]. Much attention has been given to the differential toxicity of functionalized carbon nanomaterials. In studies of functionalized and non-functionalized graphene oxide and reduced graphene oxide (up to 100 mg/L) in mussel (*Mytilus galloprovincialis*) hemocytes, nanoparticles capped with polyvinylpyrrolidone (PVP) displayed increased availability and bioaccumulation in cells leading to increased cytotoxicity [215]. 

Studies have also assessed the toxicity of functionalized MWCNTs. For example, the differential toxicity of acid oxidation (AO)-MWCNTs, polyethylene glycols (PEG)-MWCNTs, and hydroxyapatite (HA)-MWCNTs had been explored in rat bone-marrow-derived stem cells (BMSCs). At a concentration of 10 μg/mL (AO)-MWCNTs and non-functionalized MWCNTs (Raw-MWCNTs) generated significant toxicity while (PEG)-MWCNTs and (HA)-MWCNTs had minor effects and displayed favorable biocompatibility [93]. Similarly, in an in vivo study of the effects of pristine and (PEG)-MWCNTs in mice, pristine MWCNTs caused more damage to immunity than (PEG)-MWCNTs [216].

The biocompatibility of PEG coatings has also been observed in studies of SWCNTs. In human umbilical vein endothelial cells, short SWCNTs coated with long-chain pyrene-bearing polymers displayed a lower in vivo cytotoxicity over a concentration range of 5 to 100 μg/mL compared to uncoated SWCNTs [217]. Further studies have confirmed the biocompatibility of polyethylene glycol (PEG)-functionalized SWCNTs [218]. However, they also reveal further nuance in the toxicity of functionalized materials. For example, in a study of raw and PEG-SWCNTs in MDA-MB-231 cells and mice models, PEG-SWCNTs with amine terminal groups induced more toxic effects than PEG-SWCNTs with carboxyl terminal groups [218].

Attention has also been given to the differential toxicity of surface-modified metal and metal oxide particles through studies of the effects of surface capping and coating agents. For example, Niska et al. (2016) studied the in vitro toxicity of non-functionalized—uncapped (AgNPs-UC), lipoic acid-capped silver nanoparticles (AgNPs-LA), polyethylene glycol capped silver nanoparticles (AgNPs-PEG), tannic acid capped silver nanoparticles (AgNPs-TA), and silver nitrate (AgNO_3_) in human gingival fibroblast cells (HGF-1) and bacteria. They found that capping agents significantly modified the biological characteristics of silver nanoparticles. Specifically, they demonstrated that the capping agent used influences cellular toxicity. In HGF-1 cells, AgNPs-LA and AgNPs-PEG showed lesser toxic effects compared to AgNPs-UC, while in bacteria, both AgNPs-UC and AgNPs-LA showed significant toxicity [219]. Other studies and reviews have also demonstrated the effects of capping agents on toxicity [220,221,222]. 

Nanomaterials can also be fictionalized with biological elements. For example, peptide-based nanosensors have been used to detect proteases as a means of monitoring cancer [223]. Peptides used to functionalize nanomaterials will most likely degrade when released into the environment. However, due to their potential cytotoxicity, a cautionary approach is advisable to ensure that functionalized nanoparticles are highly specific and have no off-targeted toxicity. For example, peptide-coated platinum nanoparticles are very efficient in killing liver cancer cells [224]. While applying nanoparticles to kill cancer cells is beneficial, research is needed to ensure these nanoparticles will not induce toxicity in healthy cells. Furthermore, care must be taken to study the elimination and accumulation of these nanoparticles in biological systems. While combining peptide biosensors with coatings (e.g., PEG) can tune their distribution and accumulation, allowing for the application of targeted toxicity, the potential for non-specific binding and accumulation should be considered [225].

Nanomaterials are also functionalized with aptamers due to their high specificity provide a powerful tool for biosensors used in biomedical and environmental applications [226]. Aptamers are short single-stranded oligonucleotide RNA or DNA sequences, which can form secondary or 3D structures and recognize specific molecular targets. The conformation of aptamers changes after their non-covalently binding to the target molecule via hydrogen bonds, steric interactions, or van der Waals forces. For example, biosensors with aptamer functionalized nanomaterials can be used for detection of organophosphate-based pesticides. The data on the toxicity of the aptamer-functionalized nanomaterials is very limited; however, in the drug-delivery literature there is some discussion about potential off-targeted adverse effects of the aptamers such as innate immune activation and anticoagulation. At the same time other studies suggest that these effects are rare and that aptamers show very low immunogenicity but formulations (e.g., with PEG) can trigger strong immune response [227,228,229]. Some RNA aptamers were shown to cross the blood brain barrier which is beneficial for cancer therapy but might cause non-targeted effect in healthy organism though most aptamers are too large to cross the blood brain barrier. The binding of aptamers to the target molecule can also potentially result in the misfolding of the target protein.

### 3.5. Transformations

In wastewater, aquatic, or soil environments, and after uptake by organisms (in-vivo), nanomaterials can undergo various transformations resulting in modification of their surface properties, bioavailability, and toxicity [230]. Such transformations include aggregation, dissolution, oxidation and reduction, formation of biocorona, binding natural organic matter, and complexation with sulfur, phosphorus, and iron. After undergoing multiple transformations, the pristine (as manufactured) nanomaterials become “aged” nanoproducts. These aged materials differ from the original nanomaterials in their properties and behavior. Nanotoxicity studies often consider pristine nanomaterials and do not account for these transformations. However, organisms in the environment are often exposed to “aged” nanomaterials.

#### 3.5.1. Dissolution

When unstable metal or metal oxide nanoparticles are present in the aquatic environment, these nanomaterials can undergo dissolution resulting in the release of toxic ions. For example, numerous studies of AgNPs and ionic Ag show toxicity in aquatic organisms, microbes, and invertebrates at low concentrations [39,231,232]. When evaluating the toxicity of pristine nanomaterials in laboratory bench experiments, some nanomaterials, for example, ZnONP, show very rapid dissolution. Their toxicity is mainly explained by dissolution alone. In contrast, particle-specific toxicity is often observed for nanomaterials with lower dissolution rates (Ag, Co) and stable nanoparticles (Au, Pt, Pd). However, nanomaterial dissolution can also be concentration-dependent. In a study of AgNPs in a model nematode at low concentration, mortality was mainly explained by dissolution. In contrast, exposure to higher concentrations caused particle-specific toxicity [39]. The occurrence and rate of dissolution also depend on the chemistry of the exposure media selected for experiments. Therefore, one of the criteria when working with unstable nanomaterials is the inclusion of an ionic control to differentiate between particle and ionic effects under specific experimental conditions.

Adding to the complexity, in nanocomposites and nanoalloys frequently incorporated into biosensors, dissolution can be affected by interactions between metal nanoparticles (alloys) or metals and other materials (nanocomposites). For instance, when considering interactions between AgNPs and other metal nanoparticles dissolution was decreased after the formation of very stable bimetallic Au-AgNPs, while in the presence of TiO_2_NP, dissolution of AgNPs still occurred, and under dark conditions, the adverse effect of Ag-TiO_2_NPs on cell viability was similar to that of AgNPs alone. However, cell toxicity was decreased after exposure to the Ag and TiO_2_ mixture under light [233]. Thus, due to co-occurrence and interaction between metals the toxicities of the alloys can differ from their individual toxicities.

When metal nanoparticles are incorporated into 2-D nanomaterials, such as graphene, this can also influence toxicity. In a study where *Daphnia magna* was exposed to six rGO nanocomposites with different metal and metal oxide nanoparticles, exposure to rGO-Ag resulted in 15% to 36% release of Ag ions and higher toxicity across several endpoints than to rGO alone [142]. The toxicity of rGO-Ag was also higher than previously reported for commercial AgNPs in Daphnia. Since the toxicity at the total Ag concentrations without rGO was not measured, it is difficult to conclude whether the presence of rGO induced AgNPs dissolution, but certainly, it did not provide any protection. A similar result was observed for rGO-CO_3_O_4_, where toxicity was substantially higher for the nanocomposite than for rGO. However, the toxicity was lower for rGO-Pd compared to rGO alone, which was explained by the physiological effect of Pd on the daphnids resulting in their reduced uptake of rGO. A follow-up study comparing the toxicity of the six rGO nanocomposites to two species of algae demonstrated higher sensitivity for *Chlamydomonas reinhardtii* compared to *Scenedesmus obliquus*, which the authors hypothesized was due to the higher hydrophobic cell surface of the first species [141]. These findings suggest that the toxicity of nanocomposites can be affected not only by interactions among different materials but also due to their interaction with biological surfaces and by physiological responses.

#### 3.5.2. Biomolecular-Particle Complexation

In physiological environments, nanoparticles rapidly adsorb proteins, forming biomolecular-particle complexes or coronas [234]. These bound proteins interact with cell receptors and determine the cell entry mechanism and transport pathway within the cells. Thus, the corona, rather than the bare nanoparticle, is responsible for the biological response in vivo [235,236,237]. The protein corona is complex, and its composition depends on the surface morphology (size, shape, sheet structure) and chemistry (charge, capping agent, functionalization) of nanoparticles. The proteins that strongly bind to the nanoparticle surface and stay for a long-time form a” solid” protein corona, while the outer layer, where proteins can be exchanged rapidly due to a high adsorption/desorption rate, is represented by a “soft” corona. Even solid protein corona can be replaced over time.

**Figure 7:**
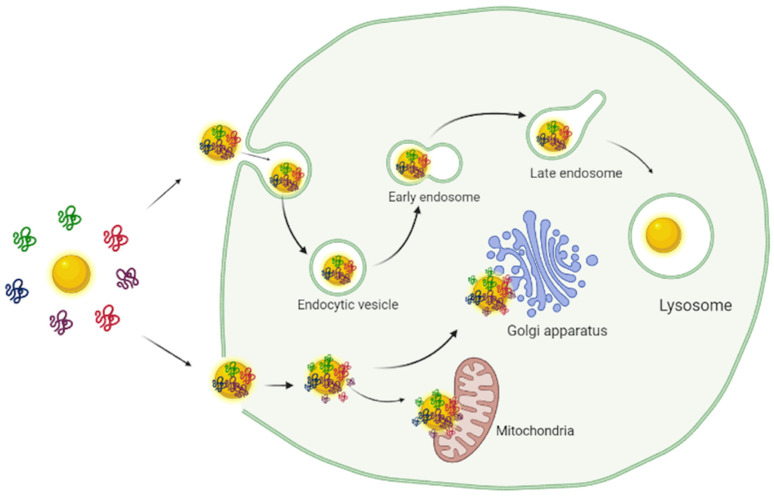
Biological fate of protein-associated nanomaterials. Protein-particle complexes can be internalized through endocytosis or translocation. Once internalized, lysosomal degradation of the corona may clear the nanomaterial of proteins, destabilizing the particles, or the particles may attach to organelles. Adapted from “Endocytic Pathway Comparison”, by BioRender.com (accessed on 2 November 2022) Retrieved from https://app.biorender.com/biorender-templates.

Some bound proteins (e.g., opsonins) and nucleic acids (e.g., aptamers) can facilitate nanoparticle entry into the cells, while others (e.g., dysopsonins) can slow it down with the nanoparticles staying longer within the circulatory system [238]. The entrance of the nanoparticles into the cells can occur via endocytosis, a more common mechanism. They can also be translocated through the cell membranes or enter due to changes in membrane curvature in the contact site with the nanoparticle surface [239]. When entry occurs through endocytosis, the nanomaterials get trapped in endosomes and eventually enter lysosomes (Figure 7). The acidic environment of lysosomes and their proteolytic enzymes cause degradation of the protein corona, ion release (for unstable particles), lysosome rupture, and lead to cell death [240]. The lysosome dysfunction is also associated with the activation of autophagy, a natural cleanup process of the damaged lysosomes, cellular debris, and misfolded proteins. If nanoparticles escape endosomes (e.g., functionalized with amines), they can be exocytosed out of the cell. Free translocation allows nanoparticles to stay bound to the inner cell membrane or directly interact with subcellular organelles and proteins. The binding of proteins to nanoparticle surfaces also can denature proteins and cause the accumulation of misfolded proteins in the cytoplasm, activation of ER stress, and unfolded protein response.

Despite the potential for protein-association leading to increased toxicity, many studies have shown that protein coronas mitigate nanomaterial toxicity. For example, the toxicity of ZnONPs decreased when associated with bovine serum albumin (BSA) when exposed to *Pseudomonas aeruginosa*, *S. aureus*, *Daphnia* sp., and *Chlorella pyrenoidosa*, due mostly to the reduction of ROS production [241]. Compared to citrate AuNPs, which were found to randomly distribute within the leaves of the fava bean plant (*Vicia faba*), BSA-coated AuNPs adhered to trichome hairs outside of the leaves rather than being internalized [242]. Additionally, in the case of 2D nanomaterials such as GO, protein coronas have been found to reduce cytotoxicity due to weakening the lipid–graphene interaction at the cell membrane, preventing particle diffusion into the cell. Overall, our understanding of the effects of protein-association on the fate and toxicity of nanomaterials is inconclusive. In vitro studies have shown increased rates of internalization and toxicity when particles are bound to proteins. Nevertheless, in vivo studies often show decreased internalization of particles, which significantly reduces the toxic potential of said nanomaterials. To better predict the environmental release of nanoparticles, it is important to consider the interactions of nanomaterials to biological components after exposure.

#### 3.5.3. Environmental Transformation and Complexation

Toxicities are often overestimated and, in some rare instances, underestimated when environmental transformations of nanomaterials are not considered. Examples of such transformations are sulfidation, complexation with phosphate, and the formation of Fe complexes. In the last decade, artificially transformed nanoparticles, such as Ag_2_S, ZnS, or Zn_2_(PO4)_3_, have been included in toxicity studies, and these transformations resulted in a substantial decrease in the nanomaterial dissolution and, thus, decreased toxicity, as observed after chronic and multigeneration exposures of *C. elegans* to transformed compared to untransformed AgNPs and ZnONPs [39,243,244]. However, even though these transformed nanomaterials are more stable, they still can be taken up by the organisms and can dissolve internally and initiate a cascade of molecular events. For example, in the Ag study, multigenerational exposure of *C. elegans* resulted in a similar accumulation of DNA mutations and epigenetic changes (histone and DNA methylation) across all treatments, including ionic Ag, pristine AgNPs, and sulfidized AgNPs [138,245]. Transformed AgNPs and ZnONPs also showed distinct transcriptomic responses compared to untransformed nanoparticles and their respective ions [243,246].

There are also studies where nanoparticle transformations occurred in biosolid-amended soils or in mesocosms. Even in a transformed, more stable form, AgNPs significantly impacted microbial community composition and function in terrestrial mesocosm study (Colman et al., 2013) and have been shown to contribute to antimicrobial resistance [247,248]. In a model legume, *Medicago truncatula*, grown in soils amended with biosolids that contain a mixture of Ag-, Ti_2_O- and ZnONP or a mixture of the respective bulk materials an adverse effect on the plant nodulation was observed only in the nanoparticle treatment. This was also supported by 200-fold and higher down-regulation of the key “nodulation” genes [249], and such plant toxicity was associated with the increase in Zn uptake. A significant shift in microbial communities was also observed in the nanoparticle treatment. In the follow-up study, with the same biosolids diluted to much lower total metal concentrations with soils, the adverse effect of the nanoparticle treatment was observed only on the microbial structure and biomass [232]. In these studies, with the mixture of three biosolid-transformed nanoparticles, ZnONPs caused toxicity to plants, while AgNPs likely also contributed to the changes in the microbial communities.

These findings raise questions about transformations and their effect on the bioavailability and toxicity of alloys and nanocomposites. For example, do the metals in nanocomposite and alloys undergo the same transformations as individual materials? Will transformations or potential degradation of 2-D nanomaterial in the nanocomposite containing AgNPs promote the release of AgNPs and their transformations? Exposure of fish larvae *Salmo trutta* to graphene oxide with a mixture of heavy metals Cr, Cu, Ni, and Zn resulted in strong sorption of the metals to the GO surface, a decrease in metal accumulation, and mitigation of the metal toxicity [250]. On the other hand, the opposite response was observed in zebrafish larvae after their co-exposure to GO and Cr^6+^, which caused a modification in the GO surface morphology and structure and enhanced toxicity [251]. Additionally, sorption of the metals to GO or other 2-D surfaces can facilitate not only metal transport in the environment but also their retention in soil and potentially their release. Photo-transformation of GO in the presence of sunlight and UV affects its physicochemical properties and can impact its mobility, sorption to natural organic matter, and, thus, its fate in the environment [252]. The complexity of the novel nanocomposites or hybrid materials for biosensing is increasing, and when such materials, containing components from two and more chemical origins, are released into the environment, it is becoming more difficult to predict their unique behavior. Will the properties of one of the components in these materials prevail, or will it be altered? Will they be more or less stable than their individual components under different environmental factors, such as pH, NOM, sunlight, and interaction with biological ligands? Many of these questions have been raised in the extensive review of hybrid materials and their environmental implications by Aich et al. (2014), and while some of these questions have been partially answered for specific nanomaterials, there is still a large gap and uncertainty about the environmental and health impacts of these nanocomposites and hybrid materials after their transformations [253].

## 4. Exposure Effects Related to Nanomaterial Life Cycles

When considering the potential environmental impact and health safety of the scaled-up production of nano biosensors, it is necessary to examine the manufacturing, utilization, and end-of-life disposal of the nanomaterials used. This type of risk assessment is considered a life cycle assessment (LCA). The life cycle includes the production of the nanomaterial-containing products, their use, disposal, and such end-of-life stages as reuse, recycling, recovery, and final disposal. The environmental impacts and health risks could be present at each stage and should be assessed [254].

At the production stage, nanomaterials can impact human health via direct occupational exposure. During the manufacturing process, individuals can be exposed to nanomaterials via the classical routes of exposure, i.e., inhalation, ingestion, and dermal contact. These exposures are of concern because, as discussed in this review, nanomaterials often display enhanced toxicity different from that of their bulk counterparts. Inhalation of nanoparticles in an occupational setting can occur via exposure to dust-containing aerosolized nanoparticles. In the workplace, aerosolized nanoparticles can be generated through several manufacturing processes [255]. Workers can be exposed through nanoproduct harvesting, processing (handlining and packaging), and equipment cleaning [256]. Once inhaled, these nanoparticles can deposit in the lungs and respiratory tract, where they have been shown to accumulate and generate toxicity [257]. Dermal exposures to nanomaterials occur through skin contact with contaminated surfaces. In an occupational setting dermal exposure to nanomaterials becomes especially problematic if nanomaterials have associated toxicities or contain impurities and if workers have compromised skin integrity [258,259].

To overcome the toxicity concerns associated with nanomaterial exposure in occupational settings greater standardization of regulations related to exposure limits and safety controls (personal and engineer) is needed. Studies have shown that if employed correctly, process modification, engineered controls, and personal protective equipment can control nanomaterial exposure. However, the implementation of these controls is often lacking, and regulatory bodies do not have many set occupational exposure limits [260,261,262]. Unless these issues are addressed the scaled-up production of biosensors will further contribute to the problem.

Potential environmental impacts associated with biosensors could also originate from the intentional and unintentional releases of nanoparticle containing waste streams (atmospheric, waste solids, and waste liquids) during production [245,246,247]. For example It has been reported that nanomaterials such as Cu, TiO_2_, Ag, or CeO_2_ could enter wastewater treatment plants (WWTPs), be eliminated mainly through the primary and secondary treatment, and then associated with the solid phases of sludge by over 80% by mass [263,264,265,266]. Dried sludge is then applied at a landfill resulting in the return of nanomaterial-contaminated sludge back to the environment. Many nanomaterials have antimicrobial properties. The initial concentration of nanomaterials present in wastewater or sludge may be low, but the accumulation of the nanomaterials in the wastewater stream and returned sludge could become problematic. It is also possible that the increased concentration of toxic nanomaterials could crash the WWTPs by killing the microorganisms essential for Biological Oxygen Demand (BOD) reduction.

Deeper understanding of the scale of environmental releases, and therefore potential environmental health concerns is limited by the lack of consistent reporting of production quantities. While some efforts are being made by organizations such as the environmental protection organization (EPA) (premanufacture notifications (PMNs) and significant new use notices (SNURs)) more robust regulations and requirements on the reporting of production are needed to assess the true risk of nanomaterial production [267].

Once products are sold to consumers further human and environmental exposures are possible. Nanomaterials are already widely applied in commercial products such as appliances, agricultural products, construction materials, cosmetics, foods, beverages, medical devices, and drug products [268,269]. As these products are used, they degrade, releasing nanomaterials into the surrounding environment [270,271]. Once in the environment these nanoparticles, which are often engineered, persist much longer due to the modifications (capping, functionalization, etc.) used to stabilize the particles [272]. In general, the utilization of biosensors for point-of-care, laboratory detection, and clinical diagnostic purposes poses limited risks. However, implantable biosensors could pose more significant risks associated with their biocompatibility and toxicity with the biosensor materials. While several countries and institutions are attempting to establish regulatory frameworks, global regulations regarding nanomaterials in consumer products are lacking [268].

Nanomaterial waste from nano biosensors can also originate from the disposal of spent devices in landfills and the leachates associated with such a disposal method. Leachates containing nanomaterials can be generated from solid waste landfills, which could directly affect both the aerobic and anaerobic processes of WWTPs. Taylor et al. investigated the impact of three copper particles, micron-, and nanoscale Cu particles, and a nanoscale Cu(OH)_2_-based fungicide on the function and operation of a model septic tank. The results indicated that systems exposed to the three Cu particles caused distinct disruptions in septic tank function. Temizel et al. (2017) studied the effect of nano-ZnO on biogas generation from simulated landfills over one year. They demonstrated up to 99% of nano-ZnO was retained in the waste matrix, leading to a decrease in biogas production of 15% [273]. Incineration is one of the important strategies for sewage sludge management. The potential for air pollution due to aerosols generated during wastewater treatment and incineration could also pose risks to the environment and human health.

To mitigate possible risks associated with the disposal of nano biosensors, methods for reuse and recycling should be explored. Traditional techniques for the recycling of nanoparticles include separation techniques such as centrifugation and solvent evaporation. However, these techniques are energy intensive. Alternative methods include the application of molecular antisolvents, pH or thermal responsive materials, and magnetic fields [274]. In batteries the successful recovery of nanomaterials has already been demonstrated at the benchtop level for nanomaterials such as Zn and ZnO nanoparticles and Graphite-polyaniline nanocomposites via Inert gas condensation (thermal) and vacuum separation, Hydrometallurgy and liquid-liquid extraction, and Oxidative polymerization and Precipitation [275]. Barriers to the effective recycling and reuse of nanomaterials arise in a lack of guidelines and strategies for the recovery and reuse of nanomaterials [274]. As researchers and regulatory bodies work to establish practical strategies and guidelines the development of reusable biosensors should be prioritized [276,277].

Strategies to reduce and control the toxicity on nanoparticles are also needed to manage the negative impacts associated with exposure. Current strategies include the coating and encapsulation, loading, grafting, and doping of nanoparticles [278,279]. The development of new strategies requires further research into the mechanisms of nanoparticle toxicity. As discussed herein nanomaterials suitable for the fabrication of biosensors have associated scales of toxicity. Greater understanding of these factors can be leveraged to develop targeted strategies to modify nanoparticles used in the fabrication of biosensors.

## 5. Conclusions

Herein we discussed toxicological factors in the context of nanomaterials used in biosensors development to foster connections between the domains of biosensors development and human and environmental toxicology. In our literature review, we discovered general agreement concerning the factors most influential to nanoparticle toxicity (material composition, dimensionality, concentration, bioaccumulation, biomagnification, surface chemistry, and transformations). However, we found little consensus on the potential negative impacts on human and environmental toxicity. While the literature on the environmental and human health effects of individual nanomaterials is plentiful, there is a lack of standardization in approaches, which we believe contributes to the heterogeneity of toxicological outcomes. For example, we observed several studies which examined the influence of one or two factors (e.g., size and shape) but failed to control for other factors, such as charge or surface modification. Furthermore, we observed variations in methodologies (e.g., administration routes, media, and model organisms) across studies. 

This lack of consensus and standardization creates a roadblock to the responsible scale-up of biosensors which must be mitigated to ensure these toxic effects are avoided. Mitigation could be achieved by adopting a holistic transdisciplinary and precautionary ethic such as the One Health approach. Broad collaboration among scientists and engineers in the domains of biosensor development, environmental toxicology, and human health is necessary to correctly identify and responsibly balance the risk of nanoparticle toxicity with the benefits of high-performance biosensors, especially in the development of biosensors that utilize alloys and nanocomposites which have received less consideration in toxicology research. Efforts such as those taken to increase comparability in studies across environmental media (aquatic testing, soil and sediment testing, biological testing, engineered systems testing, and product matrix testing media) are useful in the movement towards methodological and reporting standardization [280].

Databases describing physicochemical characteristics of nanomaterials and their environmental and human health risk and safety characteristics are available for researchers in biosensor development. Eight of these databases are well described in the recent publication by Ji et al. (2021). For example, there is caNanoLab (https://cananolab.nci.nih.gov/ accessed on 2 November 2022), for the nanomaterials used in biomedicine and this database is supported by the National Cancer Institute of the U.S. National Institutes of Health; eNanoMapper (https://data.enanomapper.net/ accessed on 2 November 2022) includes information on the nano safety assessment; there is also PubVINAS database (http://www.pubvinas.com/ accessed on 2 November 2022) for modeling of nano biological activities. The prototype of the Nanoparticle Information Library (NIL) can be found at https://www.cdc.gov/niosh/topics/nanotech (accessed on 2 November 2022) [281].

Notwithstanding the beneficial applications of nanostructured biosensors, industrial production of these materials may eventually lead to dangerous consequences if the products that contain them are managed with the conventional end-of-life approach, thus leading to persistent pollution problems analogous to the worldwide contamination with microplastics and PFAS, among others. As we work to understand the toxicity of nanoparticles utilized in biosensors, a circular approach to their development, use, and disposal are wise to limit the unmanaged human and environmental exposure of nanoparticles. 

## Figures and Tables

**Table 1:** Quantification and subdivision of Boolean query result with the keywords “biosensor *” AND “nano *’’. A total of 75,379 results were further subdivided into keyword searches.

Nanomaterial Category	Nanomaterial Keyword	Quantity of Studies Including Keyword
Nanometal	copper	3077
	gold	25,005
	platinum	2920
	silver	6625
	palladium	864
Nanometal Oxides	“copper oxide *”	352
	“iron oxide *”	1119
	“titanium dioxide *”	936
	“zinc oxide *”	1441
Carbon-Based Nanomaterials	graphene	6474
	“graphene oxide”	2783
	“carbon quantum dots”	257
	Fullerene * OR buckyball *	2946
	“carbon dot *”	709
	“carbon black nanoparticle *”	17
	“carbon nanoparticle *”	158
	“reduced graphene oxide”	2783
Composite Nanomaterials	Composite *	9650
	Alloy *	1801
Inorganic Two-Dimensional Nanomaterials	Phosphorene *	54
	“hexagonal boron nitride”	49
	“molybdenum disulfide”	512

**Table 2:** Samples of current literature on the toxicity of metal nanoparticles used in biosensors.

Size	Shape	Zeta Potential	Organism	Findings	Refs.
Copper					
10–30 nm	Spheroidal	NR ^1^	*Skeletonema costatum*	Maximum growth inhibition ratio 86% (96 h; 1.0 mg/L)	[24]
180.3–388.8 nm	NR	−12.3 mV	*Skeletonema costatum*	Maximum growth inhibition ratio 71.7% (96 h under 2 mg/L)	[25]
NR	NR	NR	*Daphnia pulex*	LC_50_/48 h = 0.5117 mg/L Increased multiplication (0.0625 mg/L and 0.125 mg/L)	[26]
40 nm	Spherical	NR	Red swamp crayfish (*Procambarus clarkii*)	LC_50_/72 h = 1.18 mg/L Decreased antioxidative enzymes activity (48 h) No significant growth inhibition or hepatopancreas alteration	[27]
50 nm	Spherical	−13 ± 2.1 to −22.8 ± 4.2 mV (15 °C) −16.2 ± 2.1 to −20.2 ± 4.7 mV (26 °C)	Juvenile rainbow trout (*Oncorhynchus mykiss*); Fathead minnow (*Pimephales promelas*); Zebrafish (*Danio rerio*)	Rainbow trout: LC_50_/96 h = 0.68 ± 0.15 mg/l; LOEC = 0.17 mg/L Fathead minnow: LC_50_/96 h = 0.28 ± 0.04 mg/L; LOEC = 0.023 mg/L Zebrafish: LC_50_/96 h = 0.22 ± 0.08 mg/L; LOEC ≤ 0.023 mg/L	[28]
<50 nm	NR	29.5 ± 0.7 mV	Rainbow trout (*Oncorhynchus mykiss*)	LC_50_/96 h = 2.00 mg/L (Acute toxicity test) Survival rate of 85.50 ± 1.33% (28 days at 0.15 mg/L) Smaller area (79.86 ± 11 μm^2^) and lower proliferation index (52.22 ± 3.26%) of hepatocytes High density of Kupffer cells	[29]
Gold					
61.69 ± 22.35 nm	Spherical	−23.50 ± 0.21 mV	*Aspergillus* *Niger; Mucor hiemalis; Penicillium chrysogenum*	Decreased survival rates at 19.697 mg/L *P. chrysogenum* least affected followed by *M. hiemalis*, then *A. niger*	[30]
50 nm	Hexagonal and round	NR	Mouse fibroblast cell line (NIH3T3); Wistar male rats	Mouse fibroblast cell line (NIH3T3): dose dependent toxicity Wistar male rats: no effects at non-toxic doses (mild changes in parts of the liver and kidney); Toxic dose induced mild changes	[31]
NR	NR	NR	*Daphnia pulex*	LC_50_/24h = 0.4027 mg/L; LC_50_/48 h = 0.1007 mg/L; 1 mg/L inhibited reproduction	[26]
10, 30, 60 nm	NR	NR	HT-29 cell lines; HepG2 cell lines; Wistar rats	AuNP in intestine, kidney, liver, spleen, feces, and urine HepG2 cell lines: decrease viability (16 h); ROS increase in all samples (10 ppm; 16 h) Wistar rats: significant rise in lipid peroxidation and protein carbonyl groups formation; No change in glucose, urea, uric acid, triglycerides, albumin, cholesterol, γ-GT, alkaline phosphatase, GOT, GPT, TNF-α, IL-1β, IL-6 and IL-10	[32]
8–28 nm (hydrodynamic radius)	Crystals	−48 to −47 mV	Wistar rats; Specific pathogen-free (SPF) Win: NMRI mice; Male Chinese; hamster lung cells; *Salmonella typhimurium* and *Escherichia coli*	Wistar rats: no unscheduled deaths, adverse clinical signs, or biologically significant differences Mice: no mortality or adverse reactions in preliminary toxicity test. No death in the main study, no adverse reactions Lung cells: No observed precipitation or relevant changes to pH or osmolality at any concentration *Salmonella typhimurium and Escherichia coli*: No increase in revertant colonies	[33]
30.5 nm	Spherical	−34.1 mV	Mix Breed Cobb chicks	Blood oxidative stress damage, histopathological alterations, and DNA fragmentation (15 ppm) Better growth performance and enhancement of final food conversion ratio (FCR) with little negative effect (5 ppm)	[34]
Platinum					
30 to 60 nm	Irregular polyhedra	NR	*Pseudo-* *kirchneriella subcapitata*	NOEC = 9.1 mg/L; EC_50_ = 16.9 mg/L; EC_100_ = 22.2 mg/L	[35]
4–9 nm	Spherical	41.2 to 0.1 mV	*Epithelioma papulosum cyprini* (EPC); Bluegill fibroblast cell line BF-2 (BF)	Dose dependent Statistically significant differences from control at 75 mg/L Impairment in cellular metabolism, including disruption of mitochondrial membrane Disruption in lysosomal integrity	[36]
5 and 70 nm	Spherical	−54.20 ± 0.44 to –11.40 ± 0.40 (5 nm particles; media dependent) –39.43 ± 0.62 to –10.04 ± 0.17 (70 nm particles; media dependent)	Neonatal mice ventricular cardio- myocytes	Decrease current densities of INa, IK1 and Ito channels but do not affect channel activity kinetics Dose-dependent decrease in heart rate Induction of complete atrioventricular conduction block (AVB) at high doses No significantly increase in ROS and leaking of lactate dehydrogenase (LDH)	[37]
20.12 nm (average)	Spherical	NR	MCF-7 cell line	Inhibited cell proliferation IC_50_/48h = 17.84 μg/mL DNA damage	[38]
Silver					
58.3 ± 12.9 nm pristine (AgNP) 64.5 ± 19.4 nm sulfidized (sAgNP)	Spherical	−6.1 mV for AgNPs −28.1 mV for sAgNPs	*Caenorhabditis elegans*	Mortality (without feeding) LC_50_: Ag ions = 7.5 μg/L; AgNP =72.5 μg/L; sAgNP = 4612 μg/L Reproduction EC_50_: Ag ions = 15.3 μg/L; AgNP= 566 μg/L; sAgNP = 4011 μg/L	[39]
2.7 and 6.5 nm	*NR*	*NR*	*Enchytraeus crypticus; FolsomiaCandida*	*Enchytraeus crypticus*: Reproduction inhibited (EC_50_/28 d = 119.3 (60.4–235.6) mg·kg/dw) for 2.7 nm; no significant effect on reproduction for 6.5 nm *Folsomia candida*: Reproduction inhibited (EC_50_/28 d = 158.7 (64.05–393.2) mg·kg/dw) for 2.7 nm; EC_50_/28d = 206.4 (181.9–234.1) mg·kg/dw for 6.5 nm No mortalities for concentrations less than or equal to 166 mg·kg/dw for 2.7 nm and 300 mg·kg/dw for 6.5 nm	[40]
100 nm	Spherical	−18.9 mV	Male Rats	Decreases in sperm motility and velocity Decreases concentrations of luteinizing hormone, follicle-stimulating hormone, and testosterone Triggered hormonal imbalance Induce oxidative stress in testis and epididymis	[41]
<100 nm	Spherical	29.5 ± 0.7 mV	Rainbow trout (*Oncorhynchus mykiss*)	LC_50_/96 h = 17.5 mg/L (Acute toxicity test) Survival rate 97.56 ± 0.33% (28 days at 1.5 mg/L) Hepatocytes show smaller area (85.22 ± 14 μm^2^) and lower proliferation index 30.37 ± 3.18%) vs. control (113.46 ± 9 μm^2^; 60.91 ± 2.21%)	[29]
10–40 nm	Spherical	NR	*Pseudokirchneriella subcapitata*	NOEC = 0.85 mg/L; EC_50_ = 1.63 mg/L; EC_100_ = 5.0 mg/L	[35]
Manufactured = 20 nm aggregation: 70.15 nm (± 21.81 nm), range of 44–95 nm	NR	−25 to −0.06 mV	Human hTERT-immortalized retinal pigmented epithelial (RPE-1) cells	Impaired cell division Further confirm toxicity to human cells Propagation of adverse phenotypes	[42]
20 nm	Spherical	*NR*	Human embryonic stem cell *(H9 hESCs)*	Significant developmental toxicity Slight effect on differentiation Disrupts specification of cranial placode Low concentrations yield no cytotoxicity or ROS generation	[43]
Palladium					
3 to 6 nm	Amorphous	−3.36 mV (negative) and 48.3 mV (positive)	Wild type adult zebrafish	Alteration of antioxidant activities Alteration of stress responses Histopathological changes Oxidative damage in the hepatic tissues	[44]
10 ± 6 nm (average)	NR	NR	Female Wistar rats	Induce immunological alterations Subchronic exposure induced decreasing trend in serum levels	[45]
Diameter ∼14 nm Thickness of ∼2 nm	Hexagonal sheets	NR	MCF-7 cells	Did not decrease the cell viability after 130 h	[46]
<10 nm	Non-uniform	NR	Human oral keratinocyte cell line RT-7	Agglomerated in membrane vesicles Suppressed proliferation Induced apoptosis Triggered the secretion of IL-1β through caspase-1 activation	[47]
3 to 15 nm	Spherical	NR	*Candida* *albicans* and *Aspergillus* *niger*	*Candida albicans*: Significant growth at 150 ppm; MIC: 212.5; LD50: 197; FC: 275; ROS increase; Cell wall damage; Nanoparticle accumulation in cell wall; Morphological changes *Aspergillus niger:* Appreciable growth inhibition at 250 mg/L; MIC: 200; FC: 250; ROS increase; Cell wall damage; Nanoparticle accumulation in cell wall; Morphological changes	[48]

^1^ NR indicated Not Reported.

**Table 3:** Samples of current literature on the toxicity of metal oxide nanoparticles used in biosensors.

Size	Shape	Zeta Potential	Organism	Findings	Refs.
Copper Oxide					
20 to 40 nm	Hexagonal	12.85 and −17.13 mV (media dependent)	Male Wistar rats	Liver function impairment Oxidative stress Inflammatory response Histopathological and ultrastructural damage ER-stress; apoptosis in liver tissue	[49]
5 nm (diameter)	Crystalline	−63.6 ± 7.9 and −13.1 ± 0.3 mV (media dependent)	Glial cells	Toxicity related to uptake of intact CuO-NPs not mediated by contaminating copper ions	[50]
50 nm	Spherical	NR ^1^	*Male Wistar rats*	Minimal changes to memory, learning performances, and locomotors activity Increased anxiety index Increased liver and stomach relative weights Slight impact on plasma biochemical parameters	[51]
34 ± 4.5 nm (Size; FESEM) 49.65 ± 2.36 nm (Diameter; TEM)	Polyhedral	1.3 mV	Indian freshwater mussel (*L. marginalis)*	Toxicity at organ and cell level Immunological and oxidative stress Decrease hemocyte count, filtration and respiration rate, and superoxide dismutase and catalase activity in hemocytes Declined phagocytosis, NO and PO activity Increased level of superoxide anion (7 and 14 d) and in activities of phosphatases	[52]
50 nm	NR	−20.72 ± 0.59 mV	*Arthrospira platensis*	Prokaryotic algae more sensitive than eukaryotic algae Damaged photosynthesis of prokaryotic algae (did not affect respiration) Copper ions release led to decline in photosynthetic capacity Accumulation of reactive oxygen species (ROS)	[53]
Iron Oxide(s)					
12 nm (average)	Spherical	NR	*Lemna minor*	Killed all leaves in 7 days (concentration nondependent) Lipid peroxidation in the plant increased (concentration dependent) Chlorophyll content decreased Iron accumulation in roots	[54]
20–40 nm	Predominantly round or spherical with few rods observed	+32.1 or +20.1 (alpha- or gamma-iron oxide)	*Selenastrum capricornutum*; *Nannochloropsis oculata*	Alpha- and gamma-iron oxide nanoparticles induce toxicity in freshwater and marine phytoplankton Adsorb to phytoplankton surface Higher concentrations had lesser effect than lower concentrations *Nannochloropsis oculata:* Gamma iron oxide nanoparticles inhibit development (54% at 0.2 mg/L) and have a high mortality rate (82%); Alpha iron oxide nanoparticles less toxic (97% mortality at 10 mg/L) *Selenastrum capricornutum:* Alpha iron oxide nanoparticles inhibit growth (73% at 0.2 mg/L); Gamma iron oxide nanoparticles inhibited growth (72% at 10 mg/L)	[55]
16 ± 2 and 7.6 ± 0.9	NR	−27.1 mV (16 nm size) and −18 mV (8 nm size)	*Chlamy-* *domonas reinhardtii*	Dose-dependent toxicity Inhibited growth Decrease in metabolic activity Increased oxidative stress Alterations in mitochondrial membrane potential Decrease in photosynthetic activity	[56]
2.3, 4.2, and 9.3 nm (diameter)	Spherical	NR	Male ICR mice	Ultrasmall (2.3 and 4.2 nm) nanoparticles highly toxic and lethal (100 mg/kg) No obvious toxicity of larger (9.3 nm) nanoparticles Control (different-sized SiO_2_ and gold nanoparticles) showed toxicity to be related to the iron element Ultrasmall nanoparticles (<5 nm) induced generation of reactive oxygen species (ROS) No obvious ROS observed with larger nanoparticles Significantly elevated ·OH levels in heart, serum, and multiple organs leading to acute cardiac failure and death	[57]
Titanium Dioxide					
56.08 nm (average)	NR	NR	Human mammary epithelial cells (MCF-7 cells)	Higher toxicity than toxicity of bulk titanium dioxide Significant reduction in viability and increased reactive oxygen species generation compared to bulk Dose and time dependent Morphological changes and retarded growth pattern (50 µg/mL dose; 12 h) Increase in apoptosis (10 µg/mL) and necrosis (50 µg/mL) compared to bulk titanium dioxide Increase in genotoxicity (12 h)	[58]
22.75 ± 7.04 nm (average diameter)	Spherical	NR	Kunming female mice	Increase titanium content in ileum Histopathological structure index of ileum significantly changed (villi height decreased and crypt depth increased) Significantly altered transcription levels of genes Apoptosis in ileum Altered intestine physical barrier (muscular layer torn and monolayer damaged) Dose-dependent	[59]
Zinc Oxide					
15.15 nm and 18.17 nm (crystalline sizes dependent on synthesis method)	Rod	NR	Testis of mature *Capra hircus*	Induce histomorphological changes (desquamation in germinal epithelium, pyknosis in germ cells, increased vacuolization, etc.) Green synthesis (*O. sanctum)* created safer nanoparticles than chemical synthesis (polyvinyl pyrrolidone (PVP)) Chemical zinc oxide nanoparticles induce cytotoxicity and apoptosis Dose and time-dependent	[60]
Thickness less than 40 nm	Sheet-like	NR	Human serum albumin (HSA); Molecular dynamics (MD) simulation	Human serum albumin (HSA): Conformational changes and intrinsic fluorescence quenched Molecular dynamics (MD) simulation: Formation of nanoparticle-protein corona and minor structural changes in protein structure	[61]
20 to 35 nm (crystalline size)	Spherical and hexagonal	NR	*Trigonella foenum graecum* L.	Biosynthesized (*Laurus nobilis)* zinc nanoparticles have a high antibacterial activity that increases with increasing contact time	[62]

^1^ NR indicated Not Reported.

**Table 4:** Summary of potential toxicity of nano metal alloys.

Nanoalloy	Atomic Percentage of Metal (x:y)	Size (nm)	Major Outcomes	References
Ag_x_: Pt_y_	10:90 to 90:10 mol% in steps of 10 mol%	15–25	Cytotoxic for human mesenchymal stem cells (hMSC) above 50 mol% silver Pt alloying inhibited Ag release and lowered cytotoxicity	[147]
Au_x_: Ag_y_	70:30 mol% 40:60 mol% 20:80 mol%	10−20	AgNP, Au_20_Ag_80_ and Au_40_Ag_60_ inhibited bacterial growth while AuNP and Au_70_Ag_30_ had little effect Au_20_Ag_80_ and AgNP decrease cell viability in mammalian cells (NIH 3T3) while AuNP, Au_70_Ag_30_, and Au_40_Ag_60_ did not. Lower cytotoxicity in mammalian cells linked to decreased Ag^+^ release	[148]
PEG coated Au_x_: Ag_y_	Not reported	<10.0	Higher biocompatibility of the coated alloy particles compared to uncoated AgNP	[149]
Ag_x_: Au_y_	20:80 wt% 80:20 wt%	~30.0 ~77.0	Eco-toxicological effects on *Daphnia Magna* in fresh water	[150]
Au_x_: Co_y_	Not known	12 ± 1.5	Alterated in gene expression, DNA damage (MNs), and DNA adduct of mice	[151]

## Data Availability

The original contributions presented in the study are included in the article/Supplementary Material; further inquiries can be directed to the corresponding author.

## Supplementary Materials

https://www.mdpi.com/article/10.3390/bios12121082/s1, Table S1. List of commonly used nanomaterials in biosensors development. Each nanoparticle category is associated with a non-comprehensive set of biosensing applications, as well as their reported properties for enhanced device performance, and detection modes.; Table S2. Studies on the toxicity of 0-D, 1-D, 2-D, and 3-D nanocarbon materials in zebra fish (*Danio rerio*) embryos.; Table S3. Studies on the size-dependent toxicity of nanometal, nanometal oxide, and nanocarbon materials used in biosensing. Studies, where smaller-sized nanomaterials induced higher toxicity, are labeled ↓ size = ↑ toxicity while studies, where larger-sized materials induce higher toxicity, are labeled ↑ size = ↑ toxicity. References [37,182,185,282,283,284,285,286,287,288,289,290,291,292,293,294,295,296,297,298,299,300,301,302,303,304,305,306,307,308,309,310,311,312,313,314,315,316,317,318,319,320,321,322,323,324,325,326,327,328,329,330,331,332,333,334,335,336,337,338,339,340,341,342,343,344,345,346,347,348,349] are cited in the supplementary material.

## References

[1] Mech A., Wohlleben W., Ghanem A., Hodoroaba V.D., Weigel S., Babick F., Brüngel R., Friedrich C.M., Rasmussen K., Rauscher H. (2020). Nano or Not Nano? A Structured Approach for Identifying Nanomaterials According to the European Commission’s Definition. Small.

[2] Khan I., Saeed K., Khan I. (2019). Nanoparticles: Properties, Applications and Toxicities. Arab. J. Chem..

[3] Sudha P.N., Sangeetha K., Vijayalakshmi K., Barhoum A. (2018). Nanomaterials History, Classification, Unique Properties, Production and Market. Emerging Applications of Nanoparticles and Architecture Nanostructures.

[4] Hammond J.L., Formisano N., Estrela P., Carrara S., Tkac J. (2016). Electrochemical Biosensors and Nanobiosensors. Essays Biochem..

[5] Luo X., Morrin A., Killard A.J., Smyth M.R. (2006). Application of Nanoparticles in Electrochemical Sensors and Biosensors. Electroanalysis.

[6] Bhalla N., Jolly P., Formisano N., Estrela P. (2016). Introduction to Biosensors. Essays Biochem..

[7] Bolotsky A., Butler D., Dong C., Gerace K., Glavin N.R., Muratore C., Robinson J.A., Ebrahimi A. (2019). Two-Dimensional Materials in Biosensing and Healthcare: From in Vitro Diagnostics to Optogenetics and Beyond. ACS Nano.

[8] Murphy L. (2006). Biosensors and Bioelectrochemistry. Curr. Opin. Chem. Biol..

[9] Kulabhusan P.K., Tripathi A., Kant K., Gold N., Kulabhusan P.K., Tripathi A., Kant K. (2022). Gold Nanoparticles and Plant Pathogens: An Overview and Prospective for Biosensing in Forestry. Sensors.

[10] Ornelas-Hernández L.F., Garduno-Robles A., Zepeda-Moreno A. (2022). A Brief Review of Carbon Dots–Silica Nanoparticles Synthesis and Their Potential Use as Biosensing and Theragnostic Applications. Nanoscale Res. Lett..

[11] Salata O.V. (2004). Applications of Nanoparticles in Biology and Medicine. J. Nanobiotechnology.

[12] Abdelbasir S.M., McCourt K.M., Lee C.M., Vanegas D.C. (2020). Waste-Derived Nanoparticles: Synthesis Approaches, Environmental Applications, and Sustainability Considerations. Front. Chem..

[13] CDC One Health Basics. https://www.cdc.gov/onehealth/basics/index.html.

[14] WHO One Health. https://www.who.int/news-room/questions-and-answers/item/one-health.

[15] Safdari M., Al-Haik M.S. (2018). A Review on Polymeric Nanocomposites: Effect of Hybridization and Synergy on Electrical Properties. Carbon-Based Polymer Nanocomposites for Environmental and Energy Applications.

[16] Ferrando R., Jellinek J., Johnston R.L. (2008). Nanoalloys: From Theory to Applications of Alloy Clusters and Nanoparticles. Chem. Rev..

[17] van Eck N.J., Waltman L. (2009). Software Survey: VOSviewer, a Computer Program for Bibliometric Mapping. Scientometrics.

[18] Chizhov A., Rumyantseva M., Gaskov A. (2021). Light Activation of Nanocrystalline Metal Oxides for Gas Sensing: Principles, Achievements, Challenges. Nanomaterials.

[19] Lim W.Y., Lan B.L., Ramakrishnan N. (2021). Emerging Biosensors to Detect Severe Acute Respiratory Syndrome Coronavirus 2 (SARS-CoV-2): A Review. Biosensors.

[20] Zhang Z., Lou Y., Guo C., Jia Q., Song Y., Tian J.Y., Zhang S., Wang M., He L., Du M. (2021). Metal–Organic Frameworks (MOFs) Based Chemosensors/Biosensors for Analysis of Food Contaminants. Trends Food Sci. Technol..

[21] Mohankumar P., Ajayan J., Mohanraj T., Yasodharan R. (2021). Recent Developments in Biosensors for Healthcare and Biomedical Applications: A Review. Measurement.

[22] Hai X., Li Y., Zhu C., Song W., Cao J., Bi S. (2020). DNA-Based Label-Free Electrochemical Biosensors: From Principles to Applications. TrAC Trends Anal. Chem..

[23] Zhang X.Q., Yin L.H., Tang M., Pu Y.P. (2011). ZnO, TiO_2_, SiO_2_, and Al_2_O_3_ Nanoparticles-Induced Toxic Effects on Human Fetal Lung Fibroblasts. Biomed. Environ. Sci..

[24] Zhang C., Chen X., Tan L., Wang J. (2018). Combined Toxicities of Copper Nanoparticles with Carbon Nanotubes on Marine Microalgae Skeletonema Costatum. Environ. Sci. Pollut. Res..

[25] Zhu X., Zhao W., Chen X., Zhao T., Tan L., Wang J. (2020). Growth Inhibition of the Microalgae Skeletonema Costatum under Copper Nanoparticles with Microplastic Exposure. Mar. Environ. Res..

[26] Garncarek M., Kowalska-Góralska M., Senze M., Czyż K. (2019). The Influence of Available Cu and Au Nanoparticles (NPs) on the Survival of Water Fleas (Daphnia Pulex). Int. J. Environ. Res. Public Health.

[27] Yang L., He Z., Li X., Jiang Z., Xuan F., Tang B., Bian X. (2022). Behavior and Toxicity Assessment of Copper Nanoparticles in Aquatic Environment: A Case Study on Red Swamp Crayfish. J. Environ. Manag..

[28] Song L., Vijver M.G., Peijnenburg W.J.G.M., Galloway T.S., Tyler C.R. (2015). A Comparative Analysis on the in Vivo Toxicity of Copper Nanoparticles in Three Species of Freshwater Fish. Chemosphere.

[29] Ostaszewska T., Śliwiński J., Kamaszewski M., Sysa P., Chojnacki M. (2018). Cytotoxicity of Silver and Copper Nanoparticles on Rainbow Trout (Oncorhynchus Mykiss) Hepatocytes. Environ. Sci. Pollut. Res..

[30] Liu K., He Z., Byrne H.J., Curtin J.F., Tian F. (2018). Investigating the Role of Gold Nanoparticle Shape and Size in Their Toxicities to Fungi. Int. J. Environ. Res. Public Health.

[31] Yahyaei B., Nouri M., Bakherad S., Hassani M., Pourali P. (2019). Effects of Biologically Produced Gold Nanoparticles: Toxicity Assessment in Different Rat Organs after Intraperitoneal Injection. AMB Express.

[32] Lopez-Chaves C., Soto-Alvaredo J., Montes-Bayon M., Bettmer J., Llopis J., Sanchez-Gonzalez C. (2018). Gold Nanoparticles: Distribution, Bioaccumulation and Toxicity. In Vitro and in Vivo Studies. Nanomed. Nanotechnol. Biol. Med..

[33] Modica V., Glávits R., Murbach T.S., Endres J.R., Hirka G., Vértesi A., Béres E., Szakonyiné I.P. (2022). A Toxicological Evaluation of 8–28 Nm Gold Nanocrystals. Food Chem. Toxicol..

[34] Hassanen E.I., Morsy E.A., Hussien A.M., Ibrahim M.A., Farroh K.Y. (2020). The Effect of Different Concentrations of Gold Nanoparticles on Growth Performance, Toxicopathological and Immunological Parameters of Broiler Chickens. Biosci. Rep..

[35] Ksiązyk M., Asztemborska M., Stęborowski R., Bystrzejewska-Piotrowska G. (2015). Toxic Effect of Silver and Platinum Nanoparticles toward the Freshwater Microalga Pseudokirchneriella Subcapitata. Bull. Environ. Contam. Toxicol..

[36] Demir V., Bucher J., Kropf C., Arenz M., Segner H. (2020). Comparative Study of Cytotoxicity by Platinum Nanoparticles and Ions in Vitro Systems Based on Fish Cell Lines. Toxicol. Vitr..

[37] Lin C.X., Gu J.L., Cao J.M. (2019). The Acute Toxic Effects of Platinum Nanoparticles on Ion Channels, Transmembrane Potentials of Cardiomyocytes in Vitro and Heart Rhythm in Vivo in Mice. Int. J. Nanomed..

[38] Şahin B., Aygün A., Gündüz H., Şahin K., Demir E., Akocak S., Şen F. (2018). Cytotoxic Effects of Platinum Nanoparticles Obtained from Pomegranate Extract by the Green Synthesis Method on the MCF-7 Cell Line. Colloids Surf. B Biointerfaces.

[39] Starnes D.L., Unrine J.M., Starnes C.P., Collin B.E., Oostveen E.K., Ma R., Lowry G.V., Bertsch P.M., Tsyusko O.V. (2015). Impact of Sulfidation on the Bioavailability and Toxicity of Silver Nanoparticles to Caenorhabditis Elegans. Environ. Pollut..

[40] Hlavkova D., Beklova M., Kopel P., Havelkova B. (2020). Effects of Silver Nanoparticles and Ions Exposure on the Soil Invertebrates Folsomia Candida and Enchytraeus Crypticus. Bull. Environ. Contam. Toxicol..

[41] Olugbodi J.O., David O., Oketa E.N., Lawal B., Okoli B.J., Mtunzi F. (2020). Silver Nanoparticles Stimulates Spermatogenesis Impairments and Hematological Alterations in Testis and Epididymis of Male Rats. Molecules.

[42] Garcia E.B., Alms C., Hinman A.W., Kelly C., Smith A., Vance M., Loncarek J., Marr L.C., Cimini D. (2019). Single-Cell Analysis Reveals That Chronic Silver Nanoparticle Exposure Induces Cell Division Defects in Human Epithelial Cells. Int. J. Environ. Res. Public Health.

[43] Hu B., Yang R., Cheng Z., Liang S., Liang S., Yin N., Faiola F. (2020). Non-Cytotoxic Silver Nanoparticle Levels Perturb Human Embryonic Stem Cell-Dependent Specification of the Cranial Placode in Part via FGF Signaling. J. Hazard. Mater..

[44] Anila P.A., Keerthiga B., Ramesh M., Muralisankar T. (2021). Synthesis and Characterization of Palladium Nanoparticles by Chemical and Green Methods: A Comparative Study on Hepatic Toxicity Using Zebrafish as an Animal Model. Comp. Biochem. Physiol. Part C Toxicol. Pharmacol..

[45] Iavicoli I., Fontana L., Leso V., Corbi M., Marinaccio A., Leopold K., Schindl R., Lucchetti D., Calapà F., Sgambato A. (2018). Subchronic Exposure to Palladium Nanoparticles Affects Serum Levels of Cytokines in Female Wistar Rats. Hum. Exp. Toxicol..

[46] Wu F.G., Jiang Y.W., Gao G., Jia H.R., Zhang X., Cheng X., Wang H.Y., Liu P. (2020). Palladium Nanosheets as Safe Radiosensitizers for Radiotherapy. Langmuir.

[47] Sasabe E., Tomomura A., Kitamura N., Yamamoto T. (2020). Metal Nanoparticles-Induced Activation of NLRP3 Inflammasome in Human Oral Keratinocytes Is a Possible Mechanism of Oral Lichenoid Lesions. Toxicol. Vitr..

[48] Athie-García M.S., Piñón-Castillo H.A., Muñoz-Castellanos L.N., Ulloa-Ogaz A.L., Martínez-Varela P.I., Quintero-Ramos A., Duran R., Murillo-Ramirez J.G., Orrantia-Borunda E. (2018). Cell Wall Damage and Oxidative Stress in Candida Albicans ATCC10231 and Aspergillus Niger Caused by Palladium Nanoparticles. Toxicol. Vitr..

[49] Liu H., Lai W., Liu X., Yang H., Fang Y., Tian L., Li K., Nie H., Zhang W., Shi Y. (2021). Exposure to Copper Oxide Nanoparticles Triggers Oxidative Stress and Endoplasmic Reticulum (ER)-Stress Induced Toxicology and Apoptosis in Male Rat Liver and BRL-3A Cell. J. Hazard. Mater..

[50] Joshi A., Thiel K., Jog K., Dringen R. (2019). Uptake of Intact Copper Oxide Nanoparticles Causes Acute Toxicity in Cultured Glial Cells. Neurochem. Res..

[51] Ouni S., Askri D., Jeljeli M., Abdelmalek H., Sakly M., Amara S. (2019). Toxicity and Effects of Copper Oxide Nanoparticles on Cognitive Performances in Rats. Arch. Environ. Occup. Health.

[52] Ray A., Gautam A., Das S., Pal K., Das S., Karmakar P., Ray M., Ray S. (2020). Effects of Copper Oxide Nanoparticle on Gill Filtration Rate, Respiration Rate, Hemocyte Associated Immune Parameters and Oxidative Status of an Indian Freshwater Mussel. Comp. Biochem. Physiol. Part C Toxicol. Pharmacol..

[53] Che X., Ding R., Zhang Q., Li Y., Sun Q., Li Y., Zhang Z., Wang W., Gao H. (2021). The Severe Toxicity of CuO Nanoparticles to the Photosynthesis of the Prokaryotic Algae Arthrospira sp.. Environ. Sci. Pollut. Res..

[54] Souza L.R.R., Bernardes L.E., Barbetta M.F.S., da Veiga M.A.M.S. (2019). Iron Oxide Nanoparticle Phytotoxicity to the Aquatic Plant Lemna Minor: Effect on Reactive Oxygen Species (ROS) Production and Chlorophyll a/Chlorophyll b Ratio. Environ. Sci. Pollut. Res. Int..

[55] Ates M., Cimen I.C.C., Unal I., Kutlu B., Ertit Tastan B., Danabas D., Aksu O., Arslan Z. (2020). Assessment of Impact of α-Fe2O3 and γ-Fe2O3 Nanoparticles on Phytoplankton Species Selenastrum Capricornutum and Nannochloropsis Oculata. Environ. Toxicol..

[56] Hurtado-Gallego J., Pulido-Reyes G., González-Pleiter M., Salas G., Leganés F., Rosal R., Fernández-Piñas F. (2020). Toxicity of Superparamagnetic Iron Oxide Nanoparticles to the Microalga Chlamydomonas Reinhardtii. Chemosphere.

[57] Wu L., Wen W., Wang X., Huang D., Cao J., Qi X., Shen S. (2022). Ultrasmall Iron Oxide Nanoparticles Cause Significant Toxicity by Specifically Inducing Acute Oxidative Stress to Multiple Organs. Part. Fibre Toxicol..

[58] Kumar S., Hussain A., Bhushan B., Kaul G. (2020). Comparative Toxicity Assessment of Nano- and Bulk-Phase Titanium Dioxide Particles on the Human Mammary Gland in Vitro. Hum. Exp. Toxicol..

[59] Yao L., Tang Y., Chen B., Hong W., Xu X., Liu Y., Aguilar Z.P., Xu H. (2020). Oral Exposure of Titanium Oxide Nanoparticles Induce Ileum Physical Barrier Dysfunction via Th1/Th2 Imbalance. Environ. Toxicol..

[60] Sharma R.K., Bareja S. (2022). Zinc Oxide Nanoparticles: Chemical and Green Synthesis, Characterization, and Comparative Evaluation of Their Effects on Caprine Testis in Vitro. J. Biochem. Mol. Toxicol..

[61] Hassanian M., Aryapour H., Goudarzi A., Javan M.B. (2020). Are Zinc Oxide Nanoparticles Safe? A Structural Study on Human Serum Albumin Using in Vitro and in Silico Methods. J. Biomol. Struct. Dyn..

[62] Chemingui H., Smiri M., Missaoui T., Hafiane A. (2019). Zinc Oxide Nanoparticles Induced Oxidative Stress and Changes in the Photosynthetic Apparatus in Fenugreek (Trigonella Foenum Graecum L.). Bull. Environ. Contam. Toxicol..

[63] Shetti N.P., Mishra A., Bukkitgar S.D., Basu S., Narang J., Raghava Reddy K., Aminabhavi T.M. (2021). Conventional and Nanotechnology-Based Sensing Methodsfor SARS Coronavirus (2019-NCoV). ACS Appl. Bio Mater..

[64] Patel R., Vinchurkar M., Patkar R., Pranjale G., Baghini M.S. Impedance Based Biosensor for Agricultural Pathogen Detection. Proceedings of the 2021 IEEE 21st International Conference on Nanotechnology (NANO).

[65] Yoon Y., Kim S., Lee J., Choi J., Kim R.K., Lee S.J., Sul O., Lee S.B. (2016). Clogging-Free Microfluidics for Continuous Size-Based Separation of Microparticles. Sci. Rep..

[66] Wasik D., Mulchandani A., Yates M.V. (2017). A Heparin-Functionalized Carbon Nanotube-Based Affinity Biosensor for Dengue Virus. Biosens. Bioelectron..

[67] Lin X., Mei Y., He C., Luo Y., Yang M., Kuang Y., Ma X., Zhang H., Huang Q. (2021). Electrochemical Biosensing Interface Based on Carbon Dots-Fe3O4 Nanomaterial for the Determination of Escherichia Coli O157:H7. Front. Chem..

[68] Wang H., Ramnan P., Pham T., Villarreal C.C., Yu X., Liu G., Mulchandani A. (2019). Gas Biosensor Arrays Based on Single-Stranded DNA-Functionalized Single-Walled Carbon Nanotubes for the Detection of Volatile Organic Compound Biomarkers Released by Huanglongbing Disease-Infected Citrus Trees. Sensors.

[69] Wan Q., Xu Y., Chen X., Xiao H. (2018). Exhaled Gas Detection by a Novel Rh-Doped CNT Biosensor for Prediagnosis of Lung Cancer: A DFT Study. Mol. Phys..

[70] Kuretake T., Kawahara S., Motooka M., Uno S. (2017). An Electrochemical Gas Biosensor Based on Enzymes Immobilized on Chromatography Paper for Ethanol Vapor Detection. Sensors.

[71] Kumar V., Raghuwanshi S.K., Kumar S. (2022). Recent Advances in Carbon Nanomaterials Based SPR Sensor for Biomolecules and Gas Detection-A Review. IEEE Sens. J..

[72] Takalkar S., Baryeh K., Liu G. (2017). Fluorescent Carbon Nanoparticle-Based Lateral Flow Biosensor for Ultrasensitive Detection of DNA. Biosens. Bioelectron..

[73] Hu F., Zhang W., Zhang J., Zhang Q., Sheng T., Gu Y. (2018). An Electrochemical Biosensor for Sensitive Detection of MicroRNAs Based on Target-Recycled Non-Enzymatic Amplification. Sens. Actuators B Chem..

[74] Bagheri S., Termehyousefi A., Mansouri N., Amani Babadi A., Abd Karim M.S., Adib Kadri N. (2017). Carbon-Based Nanobiohybrid Thin Film for Amperometric Glucose Sensing. ACS Biomater. Sci. Eng..

[75] Allafchian A.R., Moini E., Mirahmadi-Zare S.Z. (2018). Flower-Like Self-Assembly of Diphenylalanine for Electrochemical Human Growth Hormone Biosensor. IEEE Sens. J..

[76] Parate K., Pola C.C., Rangnekar S.V., Mendivelso-Perez D.L., Smith E.A., Hersam M.C., Gomes C.L., Claussen J.C. (2020). Aerosol-Jet-Printed Graphene Electrochemical Histamine Sensors for Food Safety Monitoring. 2D Mater..

[77] Nunes E.W., Silva M.K.L., Rascón J., Leiva-Tafur D., Lapa R.M.L., Cesarino I. (2022). Acetylcholinesterase Biosensor Based on Functionalized Renewable Carbon Platform for Detection of Carbaryl in Food. Biosensors.

[78] Smart A., Crew A., Pemberton R., Hughes G., Doran O., Hart J.P. (2020). Screen-Printed Carbon Based Biosensors and Their Applications in Agri-Food Safety. TrAC Trends Anal. Chem..

[79] da Silva M.K.L., Vanzela H.C., Defavari L.M., Cesarino I. (2018). Determination of Carbamate Pesticide in Food Using a Biosensor Based on Reduced Graphene Oxide and Acetylcholinesterase Enzyme. Sens. Actuators B Chem..

[80] Fallatah A., Kuperus N., Almomtan M., Padalkar S. (2022). Sensitive Biosensor Based on Shape-Controlled ZnO Nanostructures Grown on Flexible Porous Substrate for Pesticide Detection. Sensors.

[81] Zamzami M.A., Rabbani G., Ahmad A., Basalah A.A., Al-Sabban W.H., Nate Ahn S., Choudhry H. (2022). Carbon Nanotube Field-Effect Transistor (CNT-FET)-Based Biosensor for Rapid Detection of SARS-CoV-2 (COVID-19) Surface Spike Protein S1. Bioelectrochemistry.

[82] Thanihaichelvan M., Surendran S.N., Kumanan T., Sutharsini U., Ravirajan P., Valluvan R., Tharsika T. (2022). Selective and Electronic Detection of COVID-19 (Coronavirus) Using Carbon Nanotube Field Effect Transistor-Based Biosensor: A Proof-of-Concept Study. Mater. Today Proc..

[83] Saenchoopa A., Klangphukhiew S., Somsub R., Talodthaisong C., Patramanon R., Daduang J., Daduang S., Kulchat S. (2021). A Disposable Electrochemical Biosensor Based on Screen-Printed Carbon Electrodes Modified with Silver Nanowires/HPMC/Chitosan/Urease for the Detection of Mercury (II) in Water. Biosensors.

[84] Wang Z., Bi J., Wang H., Khaneghah M., Wang Z., Bi J., Wang H., Tan M. (2021). Assessment of Potential Toxicity of Onion-like Carbon Nanoparticles from Grilled Turbot Scophthalmus Maximus L.. Foods.

[85] Ou L., Song B., Liang H., Liu J., Feng X., Deng B., Sun T., Shao L. (2016). Toxicity of Graphene-Family Nanoparticles: A General Review of the Origins and Mechanisms. Part. Fibre Toxicol..

[86] Fahmi T., Branch L.D., Nima Z.A., Jang D.S., Savenka A.V., Biris A.S., Basnakian A.G. (2017). Mechanism of Graphene-Induced Cytotoxicity: Role of Endonucleases. J. Appl. Toxicol..

[87] Manjunatha B., Park S.H., Kim K., Kundapur R.R., Lee S.J. (2018). In Vivo Toxicity Evaluation of Pristine Graphene in Developing Zebrafish (Danio Rerio) Embryos. Environ. Sci. Pollut. Res..

[88] Fernandes A.L., Nascimento J.P., Santos A.P., Furtado C.A., Romano L.A., Eduardo da Rosa C., Monserrat J.M., Ventura-Lima J. (2018). Assessment of the Effects of Graphene Exposure in Danio Rerio: A Molecular, Biochemical and Histological Approach to Investigating Mechanisms of Toxicity. Chemosphere.

[89] Tiginyanu I., Ursaki V., Popa V. (2011). Ultra-Thin Membranes for Sensor Applications. Nanocoatings and Ultra-Thin Films.

[90] Khan B., Adeleye A.S., Burgess R.M., Smolowitz R., Russo S.M., Ho K.T. (2019). A 72-h Exposure Study with Eastern Oysters (Crassostrea Virginica) and the Nanomaterial Graphene Oxide. Environ. Toxicol. Chem..

[91] Khan B., Adeleye A.S., Burgess R.M., Russo S.M., Ho K.T. (2019). Effects of Graphene Oxide Nanomaterial Exposures on the Marine Bivalve, Crassostrea Virginica. Aquat. Toxicol..

[92] Souza J.P., Baretta J.F., Santos F., Paino I.M.M., Zucolotto V. (2017). Toxicological Effects of Graphene Oxide on Adult Zebrafish (Danio Rerio). Aquat. Toxicol..

[93] Zhu S., Luo F., Chen W., Zhu B., Wang G. (2017). Toxicity Evaluation of Graphene Oxide on Cysts and Three Larval Stages of Artemia Salina. Sci. Total Environ..

[94] Yu Q., Zhang B., Li J., Du T., Yi X., Li M., Chen W., Alvarez P.J.J. (2017). Graphene Oxide Significantly Inhibits Cell Growth at Sublethal Concentrations by Causing Extracellular Iron Deficiency. Nanotoxicology.

[95] Dziewięcka M., Karpeta-Kaczmarek J., Augustyniak M., Rost-Roszkowska M. (2017). Short-Term in Vivo Exposure to Graphene Oxide Can Cause Damage to the Gut and Testis. J. Hazard. Mater..

[96] An W., Zhang Y., Zhang X., Li K., Kang Y., Akhtar S., Sha X., Gao L. (2018). Ocular Toxicity of Reduced Graphene Oxide or Graphene Oxide Exposure in Mouse Eyes. Exp. Eye Res..

[97] Fadeel B., Bussy C., Merino S., Vázquez E., Flahaut E., Mouchet F., Evariste L., Gauthier L., Koivisto A.J., Vogel U. (2018). Safety Assessment of Graphene-Based Materials: Focus on Human Health and the Environment. ACS Nano.

[98] Liu X.T., Mu X.Y., Wu X.L., Meng L.X., Guan W.B., Ma Y.Q., Sun H., Wang C.J., Li X.F. (2014). Toxicity of Multi-Walled Carbon Nanotubes, Graphene Oxide, and Reduced Graphene Oxide to Zebrafish Embryos. Biomed. Environ. Sci..

[99] Xu S., Zhang Z., Chu M. (2015). Long-Term Toxicity of Reduced Graphene Oxide Nanosheets: Effects on Female Mouse Reproductive Ability and Offspring Development. Biomaterials.

[100] Guo Z., Xie C., Zhang P., Zhang J., Wang G., He X., Ma Y., Zhao B., Zhang Z. (2017). Toxicity and Transformation of Graphene Oxide and Reduced Graphene Oxide in Bacteria Biofilm. Sci. Total Environ..

[101] Siqueira P.R., Souza J.P., Estevão B.M., Altei W.F., Carmo T.L.L., Santos F.A., Araújo H.S.S., Zucolotto V., Fernandes M.N. (2022). Concentration- and Time-Dependence Toxicity of Graphene Oxide (GO) and Reduced Graphene Oxide (RGO) Nanosheets upon Zebrafish Liver Cell Line. Aquat. Toxicol..

[102] Kang Y., Liu J., Wu J., Yin Q., Liang H., Chen A., Shao L. (2017). Graphene Oxide and Reduced Graphene Oxide Induced Neural Pheochromocytoma-Derived PC12 Cell Lines Apoptosis and Cell Cycle Alterations via the ERK Signaling Pathways. Int. J. Nanomed..

[103] Wang X., Feng Y., Dong P., Huang J. (2019). A Mini Review on Carbon Quantum Dots: Preparation, Properties, and Electrocatalytic Application. Front. Chem..

[104] Sun Y., Zhang M., Bhandari B., Yang C. (2020). Recent Development of Carbon Quantum Dots: Biological Toxicity, Antibacterial Properties and Application in Foods. Food Rev. Int..

[105] Yao K., Lv X., Zheng G., Chen Z., Jiang Y., Zhu X., Wang Z., Cai Z. (2018). Effects of Carbon Quantum Dots on Aquatic Environments: Comparison of Toxicity to Organisms at Different Trophic Levels. Environ. Sci. Technol..

[106] Pinheiro F.G., Moreira-Gomes M.D., Machado M.N., dos Santos Almeida T., Barboza P.D., Silva Oliveira L.F., Ávila Cavalcante F.S., Leal-Cardoso J.H., Fortunato R.S., Zin W.A. (2021). Eugenol Mitigated Acute Lung but Not Spermatic Toxicity of C60 Fullerene Emulsion in Mice. Environ. Pollut..

[107] Sarasamma S., Audira G., Juniardi S., Sampurna B.P., Lai Y.H., Hao E., Chen J.R., Hsiao C. (2018). Der Evaluation of the Effects of Carbon 60 Nanoparticle Exposure to Adult Zebrafish: A Behavioral and Biochemical Approach to Elucidate the Mechanism of Toxicity. Int. J. Mol. Sci..

[108] Wang P., Huang B., Chen Z., Lv X., Qian W., Zhu X., Li B., Wang Z., Cai Z. (2019). Behavioural and Chronic Toxicity of Fullerene to Daphnia Magna: Mechanisms Revealed by Transcriptomic Analysis. Environ. Pollut..

[109] Zhang C., Wu L., de Perrot M., Zhao X. (2021). Carbon Nanotubes: A Summary of Beneficial and Dangerous Aspects of an Increasingly Popular Group of Nanomaterials. Front. Oncol..

[110] Venkataraman A., Amadi E.V., Chen Y., Papadopoulos C. (2019). Carbon Nanotube Assembly and Integration for Applications. Nanoscale Res. Lett..

[111] Hatami M. (2017). Toxicity Assessment of Multi-Walled Carbon Nanotubes on Cucurbita Pepo L. under Well-Watered and Water-Stressed Conditions. Ecotoxicol. Environ. Saf..

[112] Zhao J., Luo W., Xu Y., Ling J., Deng L. (2021). Potential Reproductive Toxicity of Multi-Walled Carbon Nanotubes and Their Chronic Exposure Effects on the Growth and Development of Xenopus Tropicalis. Sci. Total Environ..

[113] Deepa S., Mamta S.K., Anitha A., Senthilkumaran B. (2022). Exposure of Carbon Nanotubes Affects Testis and Brain of Common Carp. Environ. Toxicol. Pharmacol..

[114] Minchenko O.H., Tsymbal D.O., Minchenko D.O., Prylutska S.V., Hnatiuk O.S., Prylutskyy Y.I., Tsierkezos N.G., Ritter U. (2018). Single-Walled Carbon Nanotubes Affect the Expression of Genes Associated with Immune Response in Normal Human Astrocytes. Toxicol. Vitr..

[115] Lin B., Zhang H., Lin Z., Fang Y., Tian L., Yang H., Yan J., Liu H., Zhang W., Xi Z. (2013). Studies of Single-Walled Carbon Nanotubes-Induced Hepatotoxicity by NMR-Based Metabonomics of Rat Blood Plasma and Liver Extracts. Nanoscale Res. Lett..

[116] Chou C.C., Hsiao H.Y., Hong Q.S., Chen C.H., Peng Y.W., Chen H.W., Yang P.C. (2008). Single-Walled Carbon Nanotubes Can Induce Pulmonary Injury in Mouse Model. Nano Lett..

[117] Fujita K., Fukuda M., Endoh S., Maru J., Kato H., Nakamura A., Shinohara N., Uchino K., Honda K. (2016). Pulmonary and Pleural Inflammation after Intratracheal Instillation of Short Single-Walled and Multi-Walled Carbon Nanotubes. Toxicol. Lett..

[118] Zhao C., Zhou Y., Liu L., Long J., Liu H., Li J., Cao Y. (2018). Lipid Accumulation in Multi-Walled Carbon Nanotube-Exposed HepG2 Cells: Possible Role of Lipophagy Pathway. Food Chem. Toxicol..

[119] Long J., Ma W., Yu Z., Liu H., Cao Y. (2019). Multi-Walled Carbon Nanotubes (MWCNTs) Promoted Lipid Accumulation in THP-1 Macrophages through Modulation of Endoplasmic Reticulum (ER) Stress. Nanotoxicology.

[120] Yang H., Li J., Yang C., Liu H., Cao Y. (2019). Multi-Walled Carbon Nanotubes Promoted Lipid Accumulation in Human Aortic Smooth Muscle Cells. Toxicol. Appl. Pharmacol..

[121] Zhang H.Y., Chen R.L., Shao Y., Wang H.L., Liu Z.G. (2018). Effects of Exposure of Adult Mice to Multi-Walled Carbon Nanotubes on the Liver Lipid Metabolism of Their Offspring. Toxicol. Res..

[122] Zhao Y., Xu R., Hua X., Rui Q., Wang D. (2022). Multi-Walled Carbon Nanotubes Induce Transgenerational Toxicity Associated with Activation of Germline Long Non-Coding RNA Linc-7 in C. Elegans. Chemosphere.

[123] Ferreira F., Peres N.M.R., Ribeiro R.M., Chaves A.J. (2019). Excitons in Hexagonal Boron Nitride Single-Layer: A New Platform for Polaritonics in the Ultraviolet. JOSA.

[124] Mohona T.M., Gupta A., Masud A., Chien S.C., Lin L.C., Nalam P.C., Aich N. (2019). Aggregation Behavior of Inorganic 2D Nanomaterials beyond Graphene: Insights from Molecular Modeling and Modified DLVO Theory. Environ. Sci. Technol..

[125] Chng E.L.K., Pumera M. (2014). Toxicity of Graphene Related Materials and Transition Metal Dichalcogenides. RSC Adv..

[126] Naikoo G.A., Arshad F., Almas M., Hassan I.U., Pedram M.Z., Aljabali A.A., Mishra V., Serrano-Aroca Á., Birkett M., Charbe N.B. (2022). 2D Materials, Synthesis, Characterization and Toxicity: A Critical Review. Chem. Biol. Interact..

[127] Wang X., Mansukhani N.D., Guiney L.M., Ji Z., Chang C.H., Wang M., Liao Y.P., Song T.B., Sun B., Li R. (2015). Differences in the Toxicological Potential of 2D versus Aggregated Molybdenum Disulfide in the Lung. Small.

[128] Corazzari I., Deorsola F.A., Gulino G., Aldieri E., Bensaid S., Turci F., Fino D. (2014). Hazard Assessment of W and Mo Sulphide Nanomaterials for Automotive Use. J. Nanoparticle Res..

[129] Yin W., Yan L., Yu J., Tian G., Zhou L., Zheng X., Zhang X., Yong Y., Li J., Gu Z. (2014). High-Throughput Synthesis of Single-Layer MoS2 Nanosheets as a near-Infrared Photothermal-Triggered Drug Delivery for Effective Cancer Therapy. ACS Nano.

[130] Latiff N.M., Teo W.Z., Sofer Z., Fisher A.C., Pumera M. (2015). The Cytotoxicity of Layered Black Phosphorus. Chem. A Eur. J..

[131] Mu X., Wang J.Y., Bai X., Xu F., Liu H., Yang J., Jing Y., Liu L., Xue X., Dai H. (2017). Black Phosphorus Quantum Dot Induced Oxidative Stress and Toxicity in Living Cells and Mice. ACS Appl. Mater. Interfaces.

[132] Eke J., Mills P.A., Page J.R., Wright G.P., Tsyusko O.V., Escobar I.C. (2020). Nanohybrid Membrane Synthesis with Phosphorene Nanoparticles: A Study of the Addition, Stability and Toxicity. Polymers.

[133] Tian B., Tian B., Smith B., Scott M.C., Lei Q., Hua R., Tian Y., Liu Y. (2018). Facile Bottom-up Synthesis of Partially Oxidized Black Phosphorus Nanosheets as Metal-Free Photocatalyst for Hydrogen Evolution. Proc. Natl. Acad. Sci. USA.

[134] Burrs S.L., Vanegas D.C., Bhargava M., Mechulan N., Hendershot P., Yamaguchi H., Gomes C., McLamore E.S. (2015). A Comparative Study of Graphene–Hydrogel Hybrid Bionanocomposites for Biosensing. Analyst.

[135] Daniele M.A., Pedrero M., Burrs S., Chaturvedi P., Salim W.W.A., Kuralay F., Campuzano S., McLamore E., Cargill A.A., Ding S. (2015). Hybrid Metallic Nanoparticles: Enhanced Bioanalysis and Biosensing via Carbon Nanotubes, Graphene, and Organic Conjugation. Nanobiosensors Nanobioanalyses.

[136] Chaturvedi P., Vanegas D.C., Taguchi M., Burrs S.L., Sharma P., McLamore E.S. (2014). A Nanoceria–Platinum–Graphene Nanocomposite for Electrochemical Biosensing. Biosens. Bioelectron..

[137] Pacheco I., Buzea C., Kharissova O.V., Martínez L.M.T., Kharisov B.I. (2021). Nanomaterials and Nanocomposites: Classification and Toxicity. Handbook of Nanomaterials and Nanocomposites for Energy and Environmental Applications.

[138] Wamucho A., Unrine J.M., Kieran T.J., Glenn T.C., Schultz C.L., Farman M., Svendsen C., Spurgeon D.J., Tsyusko O.V. (2019). Genomic Mutations after Multigenerational Exposure of Caenorhabditis Elegans to Pristine and Sulfidized Silver Nanoparticles. Environ. Pollut..

[139] Kim Y., Jeong J., Yang J., Joo S.W., Hong J., Choi J. (2018). Graphene Oxide Nano-Bio Interaction Induces Inhibition of Spermatogenesis and Disturbance of Fatty Acid Metabolism in the Nematode Caenorhabditis Elegans. Toxicology.

[140] Tsai M.H., Chao H.R., Jiang J.J., Su Y.H., Cortez M.S.P., Tayo L.L., Lu I.C., Hsieh H., Lin C.C., Lin S.L. (2021). Toxicity of Low-Dose Graphene Oxide Nanoparticles in an in-Vivo Wild Type of Caenorhabditis Elegans Model. Aerosol Air Qual. Res..

[141] Yin J., Dong Z., Liu Y., Wang H., Li A., Zhuo Z., Feng W., Fan W. (2020). Toxicity of Reduced Graphene Oxide Modified by Metals in Microalgae: Effect of the Surface Properties of Algal Cells and Nanomaterials. Carbon N. Y..

[142] Liu Y., Fan W., Xu Z., Peng W., Luo S. (2017). Transgenerational Effects of Reduced Graphene Oxide Modified by Au, Ag, Pd, Fe_3_O_4_, Co_3_O_4_ and SnO_2_ on Two Generations of Daphnia Magna. Carbon N. Y..

[143] De Mori A., Jones R.S., Cretella M., Cerri G., Draheim R.R., Barbu E., Tozzi G., Roldo M. (2020). Evaluation of Antibacterial and Cytotoxicity Properties of Silver Nanowires and Their Composites with Carbon Nanotubes for Biomedical Applications. Int. J. Mol. Sci..

[144] Kong I.C., Ko K.S., Lee M.H., Lee J.H., Han Y.H. (2018). Ecotoxicity Evaluation of Cu- and Fe-CNT Complexes Based on the Activity of Bacterial Bioluminescence and Seed Germination. J. Environ. Sci..

[145] Hahn A., Fuhlrott J., Loos A., Barcikowski S. (2012). Cytotoxicity and Ion Release of Alloy Nanoparticles. J. Nanoparticle Res..

[146] Grade S., Eberhard J., Jakobi J., Winkel A., Stiesch M., Barcikowski S. (2014). Alloying Colloidal Silver Nanoparticles with Gold Disproportionally Controls Antibacterial and Toxic Effects. Gold Bull..

[147] Grasmik V., Breisch M., Loza K., Heggen M., Sengstock C., Epple M. (2018). Synthesis and Biological Characterization of Alloyed Silver-Platinum Nanoparticles: From Compact Core-Shell Nanoparticles to Hollow Nanoalloys. RSC Adv..

[148] Lin Z., Luo Y., Liu P., Li Y., Yue J., Jiang L. (2021). Atomic-Engineering Au-Ag Nanoalloys for Screening Antimicrobial Agents with Low Toxicity towards Mammalian Cells. Colloids Surf. B Biointerfaces.

[149] Li K., Zhao X., Zhai Y., Chen G., Lee E.H., He S. (2015). A Study on the Biocompatibility of Surface-Modified Au/Ag Alloyed Nanobox Particles in Zebrafish in Terms of Mortality Rate, Hatch Rate and Imaging of Particle Distribution Behavior. Prog. Electromagn. Res..

[150] Li T., Albee B., Alemayehu M., Diaz R., Ingham L., Kamal S., Rodriguez M., Whaley Bishnoi S. (2010). Comparative Toxicity Study of Ag, Au, and Ag-Au Bimetallic Nanoparticles on Daphnia Magna. Anal. Bioanal. Chem..

[151] Girgis E., Khalil W.K.B., Emam A.N., Mohamed M.B., Rao K.V. (2012). Nanotoxicity of Gold and Gold-Cobalt Nanoalloy. Chem. Res. Toxicol..

[152] Wang Z., Hu T., Liang R., Wei M. (2020). Application of Zero-Dimensional Nanomaterials in Biosensing. Front. Chem..

[153] Feigel I.M., Vedala H., Star A. (2011). Biosensors Based on One-Dimensional Nanostructures. J. Mater. Chem..

[154] Rohaizad N., Mayorga-Martinez C.C., Fojtů M., Latiff N.M., Pumera M. (2021). Two-Dimensional Materials in Biomedical, Biosensing and Sensing Applications. Chem. Soc. Rev..

[155] Lei Z.L., Guo B. (2022). 2D Material-Based Optical Biosensor: Status and Prospect. Adv. Sci..

[156] Napi M.L.M., Noorden A.F.A., Tan M.L.P., Jamaluddin H., Hamid F.A., Ahmad M.K., Hashim U., Ahmad M.R., Sultan S.M. (2020). Three Dimensional Zinc Oxide Nanostructures as an Active Site Platform for Biosensor: Recent Trend in Healthcare Diagnosis. J. Electrochem. Soc..

[157] Ramanathan S., Gopinath S.C.B., Arshad M.K., Poopalan P. (2019). Multidimensional (0D-3D) Nanostructures for Lung Cancer Biomarker Analysis: Comprehensive Assessment on Current Diagnostics. Biosens. Bioelectron..

[158] Raja I.S., Song S.J., Kang M.S., Lee Y.B., Kim B., Hong S.W., Jeong S.J., Lee J.C., Han D.W. (2019). Toxicity of Zero- and One-Dimensional Carbon Nanomaterials. Nanomaterials.

[159] Tan E., Li B.L., Ariga K., Lim C.T., Garaj S., Leong D.T. (2019). Toxicity of Two-Dimensional Layered Materials and Their Heterostructures. Bioconjugate Chem..

[160] Zhang B., Ni H., Chen R., Zhang T., Li X., Zhan W., Wang Z., Xu Y. (2016). Cytotoxicity Effects of Three-Dimensional Graphene in NIH-3T3 Fibroblasts. RSC Adv..

[161] Zha Y., Chai R., Song Q., Chen L., Wang X., Cheng G., Tang M., Wang M. (2016). Characterization and Toxicological Effects of Three-Dimensional Graphene Foams in Rats in Vivo. J. Nanoparticle Res..

[162] Castro P., Da C., Mayara R., Leão B., Lenz C., Corte D., Ferreira De Matos C. (2021). Evaluation of the Carbon Nanostructures Toxicity as a Function of Their Dimensionality Using Model Organisms: A Review. Water Air Soil Pollut..

[163] Jin R., Higaki T. (2021). Open Questions on the Transition between Nanoscale and Bulk Properties of Metals. Commun. Chem..

[164] Park J.H., Cho Y.W., Kim T.H. (2022). Recent Advances in Surface Plasmon Resonance Sensors for Sensitive Optical Detection of Pathogens. Biosensors.

[165] Sani A., Cao C., Cui D. (2021). Toxicity of Gold Nanoparticles (AuNPs): A Review. Biochem. Biophys. Rep..

[166] Sendra M., Moreno-Garrido I., Yeste M.P., Gatica J.M., Blasco J. (2017). Toxicity of TiO2, in Nanoparticle or Bulk Form to Freshwater and Marine Microalgae under Visible Light and UV-A Radiation. Environ. Pollut..

[167] Qiu H., Smolders E. (2017). Nanospecific Phytotoxicity of CuO Nanoparticles in Soils Disappeared When Bioavailability Factors Were Considered. Environ. Sci. Technol..

[168] Yeh Y.C., Creran B., Rotello V.M. (2012). Gold Nanoparticles: Preparation, Properties, and Applications in Bionanotechnology. Nanoscale.

[169] Bai X., Wang Y., Song Z., Feng Y., Chen Y., Zhang D., Feng L. (2020). The Basic Properties of Gold Nanoparticles and Their Applications in Tumor Diagnosis and Treatment. Int. J. Mol. Sci..

[170] Lansdown A.B.G. (2018). GOLD: Human Exposure and Update on Toxic Risks. Crit. Rev. Toxicol..

[171] Wigginton N.S., Haus K.L., Hochella M.F. (2007). Aquatic Environmental Nanoparticles. J. Environ. Monit..

[172] Huang Y.W., Cambre M., Lee H.J. (2017). The Toxicity of Nanoparticles Depends on Multiple Molecular and Physicochemical Mechanisms. Int. J. Mol. Sci..

[173] Torres-Duarte C., Ramos-Torres K.M., Rahimoff R., Cherr G.N. (2017). Stage Specific Effects of Soluble Copper and Copper Oxide Nanoparticles during Sea Urchin Embryo Development and Their Relation to Intracellular Copper Uptake. Aquat. Toxicol..

[174] Tunçsoy M., Duran S., Ay Ö., Cicik B., Erdem C. (2017). Effects of Copper Oxide Nanoparticles on Antioxidant Enzyme Activities and on Tissue Accumulation of Oreochromis Niloticus. Bull. Environ. Contam. Toxicol..

[175] Djearamane S., Lim Y.M., Wong L.S., Lee P.F. (2019). Cellular Accumulation and Cytotoxic Effects of Zinc Oxide Nanoparticles in Microalga Haematococcus Pluvialis. PeerJ.

[176] Ali I., Khan S., Shah K., Haroon, Kalimullah, Bian L. (2021). Microscopic Analysis of Plant-Mediated Silver Nanoparticle Toxicity in Rainbow Trout Fish (Oncorhynchus Mykiss). Microsc. Res. Tech..

[177] Barreto A., Luis L.G., Pinto E., Almeida A., Paíga P., Santos L.H.M.L.M., Delerue-Matos C., Trindade T., Soares A.M.V.M., Hylland K. (2019). Effects and Bioaccumulation of Gold Nanoparticles in the Gilthead Seabream (Sparus Aurata)–Single and Combined Exposures with Gemfibrozil. Chemosphere.

[178] Ickrath P., Wagner M., Scherzad A., Gehrke T., Burghartz M., Hagen R., Radeloff K., Kleinsasser N., Hackenberg S. (2017). Time-Dependent Toxic and Genotoxic Effects of Zinc Oxide Nanoparticles after Long-Term and Repetitive Exposure to Human Mesenchymal Stem Cells. Int. J. Environ. Res. Public Health.

[179] Antsiferova A.A., Kopaeva M.Y., Kochkin V.N., Kashkarov P.K., Kovalchuk M.V. (2021). Disturbance in Mammalian Cognition Caused by Accumulation of Silver in Brain. Toxics.

[180] Hara T., Saeki M., Negishi Y., Kaji T., Yamamoto C. (2020). Cell Density-Dependent Accumulation of Low Polarity Gold Nanocluster in Cultured Vascular Endothelial Cells. J. Toxicol. Sci..

[181] Wei Z., Yin X.T., Cai Y., Xu W.G., Song C.H., Wang Y.F., Zhang J.W., Kang A., Wang Z.Y., Han W. (2018). Antitumor Effect of a Pt-Loaded Nanocomposite Based on Graphene Quantum Dots Combats Hypoxia-Induced Chemoresistance of Oral Squamous Cell Carcinoma. Int. J. Nanomed..

[182] Tang H., Xu M., Zhou X.R., Zhang Y., Zhao L., Ye G., Shi F., Lv C., Li Y. (2018). Acute Toxicity and Biodistribution of Different Sized Copper Nano-Particles in Rats after Oral Administration. Mater. Sci. Eng. C.

[183] Xia Q., Huang J., Feng Q., Chen X., Liu X., Li X., Zhang T., Xiao S., Li H., Zhong Z. (2019). Size- and Cell Type-Dependent Cellular Uptake, Cytotoxicity and in Vivo Distribution of Gold Nanoparticles. Int. J. Nanomed..

[184] Baek M., Chung H.E., Yu J., Lee J.A., Kim T.H., Oh J.M., Lee W.J., Paek S.M., Lee J.K., Jeong J. (2012). Pharmacokinetics, Tissue Distribution, and Excretion of Zinc Oxide Nanoparticles. Int. J. Nanomed..

[185] Alshraiedeh N.H., Ammar O.F., Masadeh M.M., Alzoubi K.H., Al-Fandi M.G., Oweis R.J., Alsharedeh R.H., Alabed R.A., Hayajneh R.H. (2022). Comparative Study of Antibacterial Activity of Different ZnO Nanoparticles, Nanoflowers, and Nanoflakes. Curr. Nanosci..

[186] Soleimani F.F., Saleh T., Shojaosadati S.A., Poursalehi R. (2018). Green Synthesis of Different Shapes of Silver Nanostructures and Evaluation of Their Antibacterial and Cytotoxic Activity. Bionanoscience.

[187] Moon J., Kwak J., An Y.J. (2019). The Effects of Silver Nanomaterial Shape and Size on Toxicity to Caenorhabditis Elegans in Soil Media. Chemosphere.

[188] Abramenko N.B., Demidova T.B., Abkhalimov E.V., Ershov B.G., Krysanov E.Y., Kustov L.M. (2018). Ecotoxicity of Different-Shaped Silver Nanoparticles: Case of Zebrafish Embryos. J. Hazard. Mater..

[189] Sukhanova A., Bozrova S., Sokolov P., Berestovoy M., Karaulov A., Nabiev I. (2018). Dependence of Nanoparticle Toxicity on Their Physical and Chemical Properties. Nanoscale Res. Lett..

[190] Attarilar S., Yang J., Ebrahimi M., Wang Q., Liu J., Tang Y., Yang J. (2020). The Toxicity Phenomenon and the Related Occurrence in Metal and Metal Oxide Nanoparticles: A Brief Review From the Biomedical Perspective. Front. Bioeng. Biotechnol..

[191] Sree Latha T., Reddy M.C., Muthukonda S.V., Srikanth V.V.S.S., Lomada D. (2017). In Vitro and in Vivo Evaluation of Anti-Cancer Activity: Shape-Dependent Properties of TiO2 Nanostructures. Mater. Sci. Eng. C.

[192] Zein R., Sharrouf W., Selting K. (2020). Physical Properties of Nanoparticles That Result in Improved Cancer Targeting. J. Oncol..

[193] Talamini L., Violatto M.B., Cai Q., Monopoli M.P., Kantner K., Krpetić Ž., Perez-Potti A., Cookman J., Garry D., Silveira C.P. (2017). Influence of Size and Shape on the Anatomical Distribution of Endotoxin-Free Gold Nanoparticles. ACS Nano.

[194] Steckiewicz K.P., Barcinska E., Malankowska A., Zauszkiewicz–Pawlak A., Nowaczyk G., Zaleska-Medynska A., Inkielewicz-Stepniak I. (2019). Impact of Gold Nanoparticles Shape on Their Cytotoxicity against Human Osteoblast and Osteosarcoma in in Vitro Model. Evaluation of the Safety of Use and Anti-Cancer Potential. J. Mater. Sci. Mater. Med..

[195] Adams C.P., Walker K.A., Obare S.O., Docherty K.M. (2014). Size-Dependent Antimicrobial Effects of Novel Palladium Nanoparticles. PLoS ONE.

[196] Chang Y., Li K., Feng Y., Cheng Y., Zhang M., Wang Z., Wu Z., Zhang H. (2017). Achievement of Safer Palladium Nanocrystals by Enlargement of {100} Crystallographic Facets. Nanotoxicology.

[197] Ribeiro L.N.D.M., Couto V.M., Fraceto L.F., De Paula E. (2018). Use of Nanoparticle Concentration as a Tool to Understand the Structural Properties of Colloids. Sci. Rep..

[198] Abdel-Khalek A.A., Kadry M.A.M., Badran S.R., Marie M.-A.S. (2015). Comparative Toxicity of Copper Oxide Bulk and Nano Particles in Nile Tilapia; Oreochromis Niloticus: Biochemical and Oxidative Stress. J. Basic Appl. Zool..

[199] Qian H., Ke M., Qu Q., Li X., Du B., Lu T., Sun L., Pan X. (2018). Ecological Effects of Single-Walled Carbon Nanotubes on Soil Microbial Communities and Soil Fertility. Bull. Environ. Contam. Toxicol..

[200] Judy J.D., Unrine J.M., Bertsch P.M. (2011). Evidence for Biomagnification of Gold Nanoparticles within a Terrestrial Food Chain. Environ. Sci. Technol..

[201] Unrine J.M., Shoults-Wilson W.A., Zhurbich O., Bertsch P.M., Tsyusko O.V. (2012). Trophic Transfer of Au Nanoparticles from Soil along a Simulated Terrestrial Food Chain. Environ. Sci. Technol..

[202] Fröhlich E. (2012). The Role of Surface Charge in Cellular Uptake and Cytotoxicity of Medical Nanoparticles. Int. J. Nanomed..

[203] Jeon S., Clavadetscher J., Lee D.K., Chankeshwara S.V., Bradley M., Cho W.S. (2018). Surface Charge-Dependent Cellular Uptake of Polystyrene Nanoparticles. Nanomedicine.

[204] Hanot C.C., Choi Y.S., Anani T.B., Soundarrajan D., David A.E. (2015). Effects of Iron-Oxide Nanoparticle Surface Chemistry on Uptake Kinetics and Cytotoxicity in CHO-K1 Cells. Int. J. Mol. Sci..

[205] Mahmoudi M., Laurent S., Shokrgozar M.A., Hosseinkhani M. (2011). Toxicity Evaluations of Superparamagnetic Iron Oxide Nanoparticles: Cell “Vision” versus Physicochemical Properties of Nanoparticles. ACS Nano.

[206] Barbasz A., Oćwieja M., Roman M. (2017). Toxicity of Silver Nanoparticles towards Tumoral Human Cell Lines U-937 and HL-60. Colloids Surf. B Biointerfaces.

[207] Tu Z., Achazi K., Schulz A., Mülhaupt R., Thierbach S., Rühl E., Adeli M., Haag R. (2017). Combination of Surface Charge and Size Controls the Cellular Uptake of Functionalized Graphene Sheets. Adv. Funct. Mater..

[208] Weiss M., Fan J., Claudel M., Sonntag T., Didier P., Ronzani C., Lebeau L., Pons F. (2021). Density of Surface Charge Is a More Predictive Factor of the Toxicity of Cationic Carbon Nanoparticles than Zeta Potential. J. Nanobiotechnol..

[209] Li S., Wang S., Yan B., Yue T. (2021). Surface Properties of Nanoparticles Dictate Their Toxicity by Regulating Adsorption of Humic Acid Molecules. ACS Sustain. Chem. Eng..

[210] Della Ventura B., Banchelli M., Funari R., Illiano A., De Angelis M., Taroni P., Amoresano A., Matteini P., Velotta R. (2019). Biosensor Surface Functionalization by a Simple Photochemical Immobilization of Antibodies: Experimental Characterization by Mass Spectrometry and Surface Enhanced Raman Spectroscopy. Analyst.

[211] Miranda B., Rea I., Dardano P., De Stefano L., Forestiere C. (2021). Recent Advances in the Fabrication and Functionalization of Flexible Optical Biosensors: Toward Smart Life-Sciences Applications. Biosensors.

[212] Guy O.J., Walker K.A.D. (2016). Graphene Functionalization for Biosensor Applications. Silicon Carbide Biotechnology.

[213] Kumar S., Sharma R., Bhawna, Gupta A., Singh P., Kalia S., Thakur P., Kumar V. (2022). Prospects of Biosensors Based on Functionalized and Nanostructured Solitary Materials: Detection of Viral Infections and Other Risks. ACS Omega.

[214] Sanità G., Carrese B., Lamberti A. (2020). Nanoparticle Surface Functionalization: How to Improve Biocompatibility and Cellular Internalization. Front. Mol. Biosci..

[215] Katsumiti A., Tomovska R., Cajaraville M.P. (2017). Intracellular Localization and Toxicity of Graphene Oxide and Reduced Graphene Oxide Nanoplatelets to Mussel Hemocytes in Vitro. Aquat. Toxicol..

[216] Zhang T., Tang M., Zhang S., Hu Y., Li H., Zhang T., Xue Y., Pu Y. (2017). Systemic and Immunotoxicity of Pristine and PEGylated Multi-Walled Carbon Nanotubes in an Intravenous 28 Days Repeated Dose Toxicity Study. Int. J. Nanomed..

[217] Meran M., Akkus P.D., Kurkcuoglu O., Baysak E., Hizal G., Haciosmanoglu E., Unlu A., Karatepe N., Güner F.S. (2018). Noncovalent Pyrene-Polyethylene Glycol Coatings of Carbon Nanotubes Achieve in Vitro Biocompatibility. Langmuir.

[218] Shaik A.S., Shaik A.P., Bammidi V.K., Al Faraj A. (2019). Effect of Polyethylene Glycol Surface Charge Functionalization of SWCNT on the in Vitro and in Vivo Nanotoxicity and Biodistribution Monitored Noninvasively Using MRI. Toxicol. Mech. Methods.

[219] Niska K., Knap N., Kędzia A., Jaskiewicz M., Kamysz W., Inkielewicz-Stepniak I. (2016). Capping Agent-Dependent Toxicity and Antimicrobial Activity of Silver Nanoparticles: An In Vitro Study. Concerns about Potential Application in Dental Practice. Int. J. Med. Sci..

[220] Abramenko N., Demidova T.B., Krutyakov Y.A., Zherebin P.M., Krysanov E.Y., Kustov L.M., Peijnenburg W. (2019). The Effect of Capping Agents on the Toxicity of Silver Nanoparticles to Danio Rerio Embryos. Nanotoxicology.

[221] Carnovale C., Bryant G., Shukla R., Bansal V. (2019). Identifying Trends in Gold Nanoparticle Toxicity and Uptake: Size, Shape, Capping Ligand, and Biological Corona. ACS Omega.

[222] Javed R., Sajjad A., Naz S., Sajjad H., Ao Q. (2022). Significance of Capping Agents of Colloidal Nanoparticles from the Perspective of Drug and Gene Delivery, Bioimaging, and Biosensing: An Insight. Int. J. Mol. Sci..

[223] Amini A.P., Kirkpatrick J.D., Wang C.S., Jaeger A.M., Su S., Naranjo S., Zhong Q., Cabana C.M., Jacks T., Bhatia S.N. (2022). Multiscale Profiling of Protease Activity in Cancer. Nat. Commun..

[224] Shoshan M.S., Vonderach T., Hattendorf B., Wennemers H. (2019). Peptide-Coated Platinum Nanoparticles with Selective Toxicity against Liver Cancer Cells. Angew. Chem. Int. Ed..

[225] Santino F., Stavole P., He T., Pieraccini S., Paolillo M., Prodi L., Rampazzo E., Gentilucci L. (2022). Preparation of Non-Toxic Fluorescent Peptide-Coated Silica/PEG Nanoparticles from Peptide-Block Copolymer Conjugates. Micro.

[226] Kadam U.S., Hong J.C. (2022). Advances in Aptameric Biosensors Designed to Detect Toxic Contaminants from Food, Water, Human Fluids, and the Environment. Trends Environ. Anal. Chem..

[227] Kovacevic K.D., Gilbert J.C., Jilma B. (2018). Pharmacokinetics, Pharmacodynamics and Safety of Aptamers. Adv. Drug Deliv. Rev..

[228] Keefe A.D., Pai S., Ellington A. (2010). Aptamers as Therapeutics. Nat. Rev. Drug Discov..

[229] Ni S., Zhuo Z., Pan Y., Yu Y., Li F., Liu J., Wang L., Wu X., Li D., Wan Y. (2021). Recent Progress in Aptamer Discoveries and Modifications for Therapeutic Applications. ACS Appl. Mater. Interfaces.

[230] Spurgeon D.J., Lahive E., Schultz C.L. (2020). Nanomaterial Transformations in the Environment: Effects of Changing Exposure Forms on Bioaccumulation and Toxicity. Small.

[231] Fabrega J., Luoma S.N., Tyler C.R., Galloway T.S., Lead J.R. (2011). Silver Nanoparticles: Behaviour and Effects in the Aquatic Environment. Environ. Int..

[232] Chen C., Tsyusko O.V., McNear D.H., Judy J., Lewis R.W., Unrine J.M. (2017). Effects of Biosolids from a Wastewater Treatment Plant Receiving Manufactured Nanomaterials on Medicago Truncatula and Associated Soil Microbial Communities at Low Nanomaterial Concentrations. Sci. Total Environ..

[233] Sharma V.K., Sayes C.M., Guo B., Pillai S., Parsons J.G., Wang C., Yan B., Ma X. (2019). Interactions between Silver Nanoparticles and Other Metal Nanoparticles under Environmentally Relevant Conditions: A Review. Sci. Total Environ..

[234] Lundqvist M., Stigler J., Cedervall T., Berggård T., Flanagan M.B., Lynch I., Elia G., Dawson K. (2011). The Evolution of the Protein Corona around Nanoparticles: A Test Study. ACS Nano.

[235] Cukalevski R., Lundqvist M., Oslakovic C., Dahlbäck B., Linse S., Cedervall T. (2011). Structural Changes in Apolipoproteins Bound to Nanoparticles. Langmuir.

[236] Walczyk D., Bombelli F.B., Monopoli M.P., Lynch I., Dawson K.A. (2010). What the Cell “Sees” in Bionanoscience. J. Am. Chem. Soc..

[237] Fleischer C.C., Payne C.K. (2014). Secondary Structure of Corona Proteins Determines the Cell Surface Receptors Used by Nanoparticles. J. Phys. Chem. B.

[238] Breznica P., Koliqi R., Daka A. (2020). A Review of the Current Understanding of Nanoparticles Protein Corona Composition. Med. Pharm. Rep..

[239] Lin J., Miao L., Zhong G., Lin C.H., Dargazangy R., Alexander-Katz A. (2020). Understanding the Synergistic Effect of Physicochemical Properties of Nanoparticles and Their Cellular Entry Pathways. Commun. Biol..

[240] Wang F., Salvati A., Boya P. (2018). Lysosome-Dependent Cell Death and Deregulated Autophagy Induced by Amine-Modified Polystyrene Nanoparticles. Open Biol..

[241] Janani B., Raju L.L., Thomas A.M., Alyemeni M.N., Dudin G.A., Wijaya L., Alsahli A.A., Ahmad P., Khan S.S. (2021). Impact of Bovine Serum Albumin-A Protein Corona on Toxicity of ZnO NPs in Environmental Model Systems of Plant, Bacteria, Algae and Crustaceans. Chemosphere.

[242] Spielman-Sun E., Avellan A., Bland G.D., Clement E.T., Tappero R.V., Acerbo A.S., Lowry G.V. (2020). Protein Coating Composition Targets Nanoparticles to Leaf Stomata and Trichomes. Nanoscale.

[243] Starnes D., Unrine J., Chen C., Lichtenberg S., Starnes C., Svendsen C., Kille P., Morgan J., Baddar Z.E., Spear A. (2019). Toxicogenomic Responses of Caenorhabditis Elegans to Pristine and Transformed Zinc Oxide Nanoparticles. Environ. Pollut..

[244] Schultz C.L., Wamucho A., Tsyusko O.V., Unrine J.M., Crossley A., Svendsen C., Spurgeon D.J. (2016). Multigenerational Exposure to Silver Ions and Silver Nanoparticles Reveals Heightened Sensitivity and Epigenetic Memory in Caenorhabditis Elegans. Proc. R. Soc. B Biol. Sci..

[245] Wamucho A., Heffley A., Tsyusko O.V. (2020). Epigenetic Effects Induced by Silver Nanoparticles in Caenorhabditis Elegans after Multigenerational Exposure. Sci. Total Environ..

[246] Starnes D.L., Lichtenberg S.S., Unrine J.M., Starnes C.P., Oostveen E.K., Lowry G.V., Bertsch P.M., Tsyusko O.V. (2016). Distinct Transcriptomic Responses of Caenorhabditis Elegans to Pristine and Sulfidized Silver Nanoparticles. Environ. Pollut..

[247] Zhang Y., Gu A.Z., Xie S., Li X., Cen T., Li D., Chen J. (2018). Nano-Metal Oxides Induce Antimicrobial Resistance via Radical-Mediated Mutagenesis. Environ. Int..

[248] Di Cesare A., Eckert E.M., Corno G. (2016). Co-Selection of Antibiotic and Heavy Metal Resistance in Freshwater Bacteria. J. Limnol..

[249] Chen C., Unrine J.M., Judy J.D., Lewis R.W., Guo J., McNear D.H., Tsyusko O.V. (2015). Toxicogenomic Responses of the Model Legume Medicago Truncatula to Aged Biosolids Containing a Mixture of Nanomaterials (TiO_2_, Ag, and ZnO) from a Pilot Wastewater Treatment Plant. Environ. Sci. Technol..

[250] Jurgelėnė Ž., Montvydienė D., Šemčuk S., Stankevičiūtė M., Sauliutė G., Pažusienė J., Morkvėnas A., Butrimienė R., Jokšas K., Pakštas V. (2022). The Impact of Co-Treatment with Graphene Oxide and Metal Mixture on Salmo Trutta at Early Development Stages: The Sorption Capacity and Potential Toxicity. Sci. Total Environ..

[251] Chen Y., Li J., Zhou Q., Liu Z., Li Q. (2021). Hexavalent Chromium Amplifies the Developmental Toxicity of Graphene Oxide during Zebrafish Embryogenesis. Ecotoxicol. Environ. Saf..

[252] Chowdhury I., Hou W.C., Goodwin D., Henderson M., Zepp R.G., Bouchard D. (2015). Sunlight Affects Aggregation and Deposition of Graphene Oxide in the Aquatic Environment. Water Res..

[253] Aich N., Plazas-Tuttle J., Lead J.R., Saleh N.B., Aich N., Plazas-Tuttle J., Lead J.R., Saleh N.B. (2014). A Critical Review of Nanohybrids: Synthesis, Applications and Environmental Implications. Environ. Chem..

[254] Donia D.T., Carbone M. (2018). Fate of the Nanoparticles in Environmental Cycles. Int. J. Environ. Sci. Technol..

[255] Ferdous Z., Nemmar A. (2020). Health Impact of Silver Nanoparticles: A Review of the Biodistribution and Toxicity Following Various Routes of Exposure. Int. J. Mol. Sci..

[256] Heitbrink W.A., Lo L.M., Dunn K.H. (2015). Exposure Controls for Nanomaterials at Three Manufacturing Sites. J. Occup. Environ. Hyg..

[257] Xia T., Li N., Nel A.E. (2009). Potential Health Impact of Nanoparticles. Annu. Rev. Public Health.

[258] Larese Filon F., Bello D., Cherrie J.W., Sleeuwenhoek A., Spaan S., Brouwer D.H. (2016). Occupational Dermal Exposure to Nanoparticles and Nano-Enabled Products: Part I—Factors Affecting Skin Absorption. Int. J. Hyg. Environ. Health.

[259] Goede H., Christopher-De Vries Y., Kuijpers E., Fransman W. (2018). A Review of Workplace Risk Management Measures for Nanomaterials to Mitigate Inhalation and Dermal Exposure. Ann. Work Expo. Health.

[260] Kim J., Yu I.J. (2016). National Survey of Workplaces Handling and Manufacturing Nanomaterials, Exposure to and Health Effects of Nanomaterials, and Evaluation of Nanomaterial Safety Data Sheets. Biomed Res. Int..

[261] Methner M., Hodson L., Dames A., Geraci C. (2010). Nanoparticle Emission Assessment Technique (NEAT) for the Identification and Measurement of Potential Inhalation Exposure to Engineered Nanomaterials—Part B: Results from 12 Field Studies. J. Occup. Environ. Hyg..

[262] OSHA (2013). What Are Nanotechnology and Nanomaterials?.

[263] Ganesh R., Smeraldi J., Hosseini T., Khatib L., Olson B.H., Rosso D. (2010). Evaluation of Nanocopper Removal and Toxicity in Municipal Wastewaters. Environ. Sci. Technol..

[264] Gómez-Rivera F., Field J.A., Brown D., Sierra-Alvarez R. (2012). Fate of Cerium Dioxide (CeO_2_) Nanoparticles in Municipal Wastewater during Activated Sludge Treatment. Bioresour. Technol..

[265] Kaegi R., Voegelin A., Sinnet B., Zuleeg S., Hagendorfer H., Burkhardt M., Siegrist H. (2011). Behavior of Metallic Silver Nanoparticles in a Pilot Wastewater Treatment Plant. Environ. Sci. Technol..

[266] Wang Y., Westerhoff P., Hristovski K.D. (2012). Fate and Biological Effects of Silver, Titanium Dioxide, and C60 (Fullerene) Nanomaterials during Simulated Wastewater Treatment Processes. J. Hazard. Mater..

[267] Hendren C.O., Mesnard X., Dröge J., Wiesner M.R. (2011). Estimating Production Data for Five Engineered Nanomaterials as a Basis for Exposure Assessment. Environ. Sci. Technol..

[268] Subhan M.A., Subhan T. (2022). Safety and Global Regulations for Application of Nanomaterials. Nanomaterials Recycling.

[269] Food and Drug Administration, Office of the Commissioner, Office of Policy, L.I.A., Office of Policy (2014). Considering Whether an FDA-Regulated Product Involves the Application of Nanotechnology.

[270] Yang Y., Westerhoff P. (2014). Presence in, and Release of, Nanomaterials from Consumer Products. Adv. Exp. Med. Biol..

[271] Malakar A., Kanel S.R., Ray C., Snow D.D., Nadagouda M.N. (2021). Nanomaterials in the Environment, Human Exposure Pathway, and Health Effects: A Review. Sci. Total Environ..

[272] Bhatt I., Tripathi B.N. (2011). Interaction of Engineered Nanoparticles with Various Components of the Environment and Possible Strategies for Their Risk Assessment. Chemosphere.

[273] Temizel İ., Emadian S.M., Di Addario M., Onay T.T., Demirel B., Copty N.K., Karanfil T. (2017). Effect of Nano-ZnO on Biogas Generation from Simulated Landfills. Waste Manag..

[274] Živković D., Balanović L., Mitovski A., Talijan N., Štrbac N., Sokić M., Manasijević D., Minić D., Ćosović V. (2015). Nanomaterials Environmental Risks and Recycling: Actual Issues. Reciklaza I Odrziv. Razvoj.

[275] Pavoski G., Botelho Junior A.B., Chaves R.M., Maraschin T., Oviedo L.R., Martins T.A.G., da Silva W.L., Bertuol D.A., Espinosa D.C.R. (2022). Nanotechnology and Recycling, Remanufacturing, and Reusing Battery. Nanotechnology and Recycling, Remanufacturing, and Reusing Battery.

[276] Hwang C., Park N., Kim E.S., Kim M., Kim S.D., Park S., Kim N.Y., Kim J.H. (2021). Ultra-Fast and Recyclable DNA Biosensor for Point-of-Care Detection of SARS-CoV-2 (COVID-19). Biosens. Bioelectron..

[277] Shi L., Wang Y., Chu Z., Yin Y., Jiang D., Luo J., Ding S., Jin W. (2017). A Highly Sensitive and Reusable Electrochemical Mercury Biosensor Based on Tunable Vertical Single-Walled Carbon Nanotubes and a Target Recycling Strategy. J. Mater. Chem. B.

[278] Yan L., Zhao F., Wang J., Zu Y., Gu Z., Zhao Y. (2019). A Safe-by-Design Strategy towards Safer Nanomaterials in Nanomedicines. Adv. Mater..

[279] Najahi-Missaoui W., Arnold R.D., Cummings B.S. (2020). Safe Nanoparticles: Are We There Yet?. Int. J. Mol. Sci..

[280] Geitner N.K., Ogilvie Hendren C., Cornelis G., Kaegi R., Lead J.R., Lowry G.V., Lynch I., Nowack B., Petersen E., Bernhardt E. (2020). Harmonizing across Environmental Nanomaterial Testing Media for Increased Comparability of Nanomaterial Datasets. Environ. Sci. Nano.

[281] Ji Z., Guo W., Sakkiah S., Liu J., Patterson T.A., Hong H. (2021). Nanomaterial Databases: Data Sources for Promoting Design and Risk Assessment of Nanomaterials. Nanomaterials.

[282] Li J., Si L., Bao J., Wang Z., Dai Z. (2017). Fluorescence Regulation of Poly(Thymine)-Templated Copper Nanoparticles via an Enzyme-Triggered Reaction toward Sensitive and Selective Detection of Alkaline Phosphatase. Anal. Chem..

[283] Bogers J.F.M., Berghuis N.F., Busker R.W., Van Booma A., Paauw A., Van Leeuwen H.C. (2021). Bright Fluorescent Nucleic Acid Detection with CRISPR-Cas12a and Poly(Thymine) Templated Copper Nanoparticles. Biol. Methods Protoc..

[284] Wang Z., Han P., Mao X., Yin Y., Cao Y. (2017). Sensitive Detection of Glutathione by Using DNA-Templated Copper Nanoparticles as Electrochemical Reporters. Sens. Actuators B Chem..

[285] Bai J., Jiang X. (2013). A Facile One-Pot Synthesis of Copper Sulfide-Decorated Reduced Graphene Oxide Composites for Enhanced Detecting of H_2_O_2_ in Biological Environments. Anal. Chem..

[286] Hussein H.A., El Nashar R.M., El-Sherbiny I.M., Hassan R.Y.A. (2021). High Selectivity Detection of FMDV- SAT-2 Using a Newly-Developed Electrochemical Nanosensors. Biosens. Bioelectron..

[287] Anh N.T., Dinh N.X., Van Tuan H., Doan M.Q., Anh N.H., Khi N.T., Trang V.T., Tri D.Q., Le A.T. (2022). Eco-Friendly Copper Nanomaterials-Based Dual-Mode Optical Nanosensors for Ultrasensitive Trace Determination of Amoxicillin Antibiotics Residue in Tap Water Samples. Mater. Res. Bull..

[288] Qing Z., Bai A., Xing S., Zou Z., He X., Wang K., Yang R. (2019). Progress in Biosensor Based on DNA-Templated Copper Nanoparticles. Biosens. Bioelectron..

[289] Mokhtarzadeh A., Eivazzadeh-Keihan R., Pashazadeh P., Hejazi M., Gharaatifar N., Hasanzadeh M., Baradaran B., de la Guardia M. (2017). Nanomaterial-Based Biosensors for Detection of Pathogenic Virus. TrAC Trends Anal. Chem..

[290] Stebunov Y.V., Yakubovsky D.I., Fedyanin D.Y., Arsenin A.V., Volkov V.S. (2018). Superior Sensitivity of Copper-Based Plasmonic Biosensors. Langmuir.

[291] Azimzadeh M., Rahaie M., Nasirizadeh N., Ashtari K., Naderi-Manesh H. (2016). An Electrochemical Nanobiosensor for Plasma MiRNA-155, Based on Graphene Oxide and Gold Nanorod, for Early Detection of Breast Cancer. Biosens. Bioelectron..

[292] Ramesh T., Foo K.L., Haarindraprasad R., Sam A.J., Solayappan M. (2019). Gold-Hybridized Zinc Oxide Nanorods as Real-Time Low-Cost NanoBiosensors for Detection of Virulent DNA Signature of HPV-16 in Cervical Carcinoma. Sci. Rep..

[293] Faridli Z., Mahani M., Torkzadeh-Mahani M., Fasihi J. (2016). Development of a Localized Surface Plasmon Resonance-Based Gold Nanobiosensor for the Determination of Prolactin Hormone in Human Serum. Anal. Biochem..

[294] Vakili S., Samare-Najaf M., Dehghanian A., Tajbakhsh A., Askari H., Tabrizi R., Iravani Saadi M., Movahedpour A., Alizadeh M., Samareh A. (2021). Gold Nanobiosensor Based on the Localized Surface Plasmon Resonance Is Able to Diagnose Human Brucellosis, Introducing a Rapid and Affordable Method. Nanoscale Res. Lett..

[295] Salahvarzi A., Mahani M., Torkzadeh-Mahani M., Alizadeh R. (2017). Localized Surface Plasmon Resonance Based Gold Nanobiosensor: Determination of Thyroid Stimulating Hormone. Anal. Biochem..

[296] Ying N., Ju C., Li Z., Liu W., Wan J. (2017). Visual Detection of Nucleic Acids Based on Lateral Flow Biosensor and Hybridization Chain Reaction Amplification. Talanta.

[297] Zheng L., Cai G., Wang S., Liao M., Li Y., Lin J. (2019). A Microfluidic Colorimetric Biosensor for Rapid Detection of Escherichia Coli O157:H7 Using Gold Nanoparticle Aggregation and Smart Phone Imaging. Biosens. Bioelectron..

[298] Elahi N., Kamali M., Baghersad M.H., Amini B. (2019). A Fluorescence Nano-Biosensors Immobilization on Iron (MNPs) and Gold (AuNPs) Nanoparticles for Detection of Shigella spp.. Mater. Sci. Eng. C.

[299] Hosseini M., Ahmadi E., Borghei Y.S., Ganjali M.R. (2017). A New Fluorescence Turn-on Nanobiosensor for the Detection of Micro-RNA-21 Based on a DNA–Gold Nanocluster. Methods Appl. Fluoresc..

[300] Tessaro L., Aquino A., de Carvalho A.P.A., Conte-Junior C.A. (2021). A Systematic Review on Gold Nanoparticles Based-Optical Biosensors for Influenza Virus Detection. Sens. Actuators Rep..

[301] Ma X.M., Sun M., Lin Y., Liu Y.J., Luo F., Guo L.H., Qiu B., Lin Z.Y., Chen G.N. (2018). Progress of Visual Biosensor Based on Gold Nanoparticles. Chin. J. Anal. Chem..

[302] Sharifi M., Hosseinali S.H., Hossein Alizadeh R., Hasan A., Attar F., Salihi A., Shekha M.S., Amen K.M., Aziz F.M., Saboury A.A. (2020). Plasmonic and Chiroplasmonic Nanobiosensors Based on Gold Nanoparticles. Talanta.

[303] Proa-Coronado S., Vargas-García J.R., Manzo-Robledo A., Mendoza-Acevedo S., Villagómez C.J., Mercado-Zúñiga C., Muñoz-Aguirre N., Villa-Vargas L.A., Martinez-Rivas A. (2018). Platinum Nanoparticles Homogenously Decorating Multilayered Reduced Graphene Oxide for Electrical Nanobiosensor Applications. Thin Solid Film..

[304] Dash S.R., Bag S.S., Golder A.K. (2021). Bio-Inspired PtNPs/Graphene Nanocomposite Based Electrocatalytic Sensing of Metabolites of Dipyrone. Anal. Chim. Acta.

[305] Unmüssig T., Weltin A., Urban S., Daubinger P., Urban G.A., Kieninger J. (2018). Non-Enzymatic Glucose Sensing Based on Hierarchical Platinum Micro-/Nanostructures. J. Electroanal. Chem..

[306] You J.G., Liu Y.W., Lu C.Y., Tseng W.L., Yu C.J. (2017). Colorimetric Assay of Heparin in Plasma Based on the Inhibition of Oxidase-like Activity of Citrate-Capped Platinum Nanoparticles. Biosens. Bioelectron..

[307] Deng H.H., Lin X.L., Liu Y.H., Li K.L., Zhuang Q.Q., Peng H.P., Liu A.L., Xia X.H., Chen W. (2017). Chitosan-Stabilized Platinum Nanoparticles as Effective Oxidase Mimics for Colorimetric Detection of Acid Phosphatase. Nanoscale.

[308] Bagheri Pebdeni A., Hosseini M. (2020). Fast and Selective Whole Cell Detection of Staphylococcus Aureus Bacteria in Food Samples by Paper Based Colorimetric Nanobiosensor Using Peroxidase-like Catalytic Activity of DNA-Au/Pt Bimetallic Nanoclusters. Microchem. J..

[309] Rithesh Raj D., Prasanth S., Vineeshkumar T.V., Sudarsanakumar C. (2016). Surface Plasmon Resonance Based Fiber Optic Dopamine Sensor Using Green Synthesized Silver Nanoparticles. Sens. Actuators B Chem..

[310] Bhalla N., Jamshaid A., Leung M.H.M., Ishizu N., Shen A.Q. (2019). Electrical Contact of Metals at the Nanoscale Overcomes the Oxidative Susceptibility of Silver-Based Nanobiosensors. ACS Appl. Nano Mater..

[311] Vasileva P., Donkova B., Karadjova I., Dushkin C. (2011). Synthesis of Starch-Stabilized Silver Nanoparticles and Their Application as a Surface Plasmon Resonance-Based Sensor of Hydrogen Peroxide. Colloids Surf. A Physicochem. Eng. Asp..

[312] Zhao L.J., Yu R.J., Ma W., Han H.X., Tian H., Qian R.C., Long Y.T. (2017). Sensitive Detection of Protein Biomarkers Using Silver Nanoparticles Enhanced Immunofluorescence Assay. Theranostics.

[313] Mansourian N., Rahaie M., Hosseini M. (2017). A Nanobiosensor Based on Fluorescent DNA-Hosted Silver Nanocluster and HCR Amplification for Detection of MicroRNA Involved in Progression of Multiple Sclerosis. J. Fluoresc..

[314] Ajay R.F.I., Tshoko S., Mgwili Y., Nqunqa S., Mulaudzi T., Mayedwa N., Iwuoha E. (2020). Green Method Synthesised Graphene-Silver Electrochemical Nanobiosensors for Ethambutol and Pyrazinamide. Processes.

[315] Yi X., Wu Y., Tan G., Yu P., Zhou L., Zhou Z., Chen J., Wang Z., Pang J., Ning C. (2017). Palladium Nanoparticles Entrapped in a Self-Supporting Nanoporous Gold Wire as Sensitive Dopamine Biosensor. Sci. Rep..

[316] Silina Y.E., Apushkinskaya N., Talagaeva N.V., Levchenko M.G., Zolotukhina E.V. (2021). Electrochemical Operational Principles and Analytical Performance of Pd-Based Amperometric Nanobiosensors. Analyst.

[317] Ma J., Zhou Y., Bai X., Chen K., Guan B.O. (2019). High-Sensitivity and Fast-Response Fiber-Tip Fabry–Pérot Hydrogen Sensor with Suspended Palladium-Decorated Graphene. Nanoscale.

[318] Huang X., Zhu Y., Kianfar E. (2021). Nano Biosensors: Properties, Applications and Electrochemical Techniques. J. Mater. Res. Technol..

[319] Butyrskaya E.V., Korkmaz N., Zolotukhina E.V., Krasiukova V., Silina Y.E. (2021). Mechanistic Aspects of Functional Layer Formation in Hybrid One-Step Designed GOx/Nafion/Pd-NPs Nanobiosensors. Analyst.

[320] Dhiman T.K., Lakshmi G.B.V.S., Kumar R., Asokan K., Solanki P.R. (2020). Non-Enzymatic Detection of Glucose Using a Capacitive Nanobiosensor Based on PVA Capped CuO Synthesized via Co-Precipitation Route. IEEE Sens. J..

[321] Roohizadeh A., Ghaffarinejad A., Salahandish R., Omidinia E. (2020). Label-Free RNA-Based Electrochemical Nanobiosensor for Detection of Hepatitis C. Curr. Res. Biotechnol..

[322] Bao J., Huang T., Wang Z., Yang H., Geng X., Xu G., Samalo M., Sakinati M., Huo D., Hou C. (2019). 3D Graphene/Copper Oxide Nano-Flowers Based Acetylcholinesterase Biosensor for Sensitive Detection of Organophosphate Pesticides. Sens. Actuators B Chem..

[323] Yang Z., Yi C., Lv S., Sheng Y., Wen W., Zhang X., Wang S. (2019). Development of a Lateral Flow Strip Biosensor Based on Copper Oxide Nanoparticles for Rapid and Sensitive Detection of HPV16 DNA. Sens. Actuators B Chem..

[324] Ain N.U., Safdar N., Yasmin A. (2019). Additive-Based Stability Assessment of Biologically Designed CuO and GSH-CuO Nanospheres and Their Applicability as Nano-Biosensors. Colloids Surf. B Biointerfaces.

[325] George J.M., Antony A., Mathew B. (2018). Metal Oxide Nanoparticles in Electrochemical Sensing and Biosensing: A Review. Microchim. Acta.

[326] Abbasi A., Ghorban K., Nojoomi F., Dadmanesh M. (2021). Smaller Copper Oxide Nanoparticles Have More Biological Effects Versus Breast Cancer and Nosocomial Infections Bacteria. Asian Pac. J. Cancer Prev..

[327] Jin L., Li T., Yang T., Liang X., Wu B., Zou D., Hu L., Huang G., Zhang J. (2020). NMR Rapid Detection of Salmonella in Milk Based on Ultra-Small Iron Oxide Nanobiosensor. Int. Dairy J..

[328] 25Waifalkar P.P., Chougale A.D., Kollu P., Patil P.S., Patil P.B. (2018). Magnetic Nanoparticle Decorated Graphene Based Electrochemical Nanobiosensor for H2O2 Sensing Using HRP. Colloids Surf. B Biointerfaces.

[329] Kumar S., Umar M., Saifi A., Kumar S., Augustine S., Srivastava S., Malhotra B.D. (2019). Electrochemical Paper Based Cancer Biosensor Using Iron Oxide Nanoparticles Decorated PEDOT:PSS. Anal. Chim. Acta.

[330] Malalasekera A.P., Wang H., Samarakoon T.N., Udukala D.N., Yapa A.S., Ortega R., Shrestha T.B., Alshetaiwi H., McLaurin E.J., Troyer D.L. (2017). A Nanobiosensor for the Detection of Arginase Activity. Nanomed. Nanotechnol. Biol. Med..

[331] Elahi N., Rizwan M. (2021). Progress and Prospects of Magnetic Iron Oxide Nanoparticles in Biomedical Applications: A Review. Artif. Organs.

[332] Nadzirah S., Hashim U., Gopinath S.C.B., Parmin N.A., Hamzah A.A., Yu H.W., Dee C.F. (2020). Titanium Dioxide–Mediated Resistive Nanobiosensor for E. Coli O157:H7. Microchim. Acta.

[333] Parmin N.A., Hashim U., Gopinath S.C.B., Nadzirah S., Rejali Z., Afzan A., Uda M.N.A., Hong V.C., Rajapaksha R.D.A.A. (2019). Voltammetric Determination of Human Papillomavirus 16 DNA by Using Interdigitated Electrodes Modified with Titanium Dioxide Nanoparticles. Microchim. Acta.

[334] Zani V., Pedron D., Pilot R., Signorini R. (2020). Biocompatible Temperature Nanosensors Based on Titanium Dioxide. Multidiscip. Digit. Publ. Inst. Proc..

[335] Paul K.B., Vanjari S.R.K., Singh S.G. (2017). Highly Sensitive Electrospun Multiwalled Carbon Nanotubes Embedded Zinc Oxide Nanowire Based Interface for Label Free Biosensing. Procedia Technol..

[336] Li X., Qin Z., Fu H., Li T., Peng R., Li Z., Rini J.M., Liu X. (2021). Enhancing the Performance of Paper-Based Electrochemical Impedance Spectroscopy Nanobiosensors: An Experimental Approach. Biosens. Bioelectron..

[337] Barbillon G. (2019). Fabrication and SERS Performances of Metal/Si and Metal/ZnO Nanosensors: A Review. Coatings.

[338] Usenko C.Y., Harper S.L., Tanguay R.L. (2007). In Vivo Evaluation of Carbon Fullerene Toxicity Using Embryonic Zebrafish. Carbon N. Y..

[339] Chen L.Q., Hu P.P., Zhang L., Huang S.Z., Luo L.F., Huang C.Z. (2012). Toxicity of Graphene Oxide and Multi-Walled Carbon Nanotubes against Human Cells and Zebrafish. Sci. China Chem..

[340] d’Amora M., Lamberti A., Fontana M., Giordani S. (2020). Toxicity Assessment of Laser-Induced Graphene by Zebrafish during Development. J. Phys. Mater..

[341] Fernández M.N., Muñoz-Olivas R., Luque-Garcia J.L. (2019). SILAC-Based Quantitative Proteomics Identifies Size-Dependent Molecular Mechanisms Involved in Silver Nanoparticles-Induced Toxicity. Nanotoxicology.

[342] Wongrakpanich A., Mudunkotuwa I.A., Geary S.M., Morris A.S., Mapuskar K.A., Spitz D.R., Grassian V.H., Salem A.K. (2016). Size-Dependent Cytotoxicity of Copper Oxide Nanoparticles in Lung Epithelial Cells. Environ. Sci. Nano.

[343] Ying H., Ruan Y., Zeng Z., Bai Y., Xu J., Chen S. (2022). Iron Oxide Nanoparticles Size-Dependently Activate Mouse Primary Macrophages via Oxidative Stress and Endoplasmic Reticulum Stress. Int. Immunopharmacol..

[344] Liao F., Chen L., Liu Y., Zhao D., Peng W., Wang W., Feng S. (2019). The Size-Dependent Genotoxic Potentials of Titanium Dioxide Nanoparticles to Endothelial Cells. Environ. Toxicol..

[345] Wang B., Zhang J., Chen C., Xu G., Qin X., Hong Y., Bose D.D., Qiu F., Zou Z. (2018). The Size of Zinc Oxide Nanoparticles Controls Its Toxicity through Impairing Autophagic Flux in A549 Lung Epithelial Cells. Toxicol. Lett..

[346] Jia P.P., Sun T., Junaid M., Yang L., Ma Y.B., Cui Z.S., Wei D.P., Shi H.F., Pei D.S. (2019). Nanotoxicity of Different Sizes of Graphene (G) and Graphene Oxide (GO) in Vitro and in Vivo. Environ. Pollut..

[347] Luo Y., Peng J., Huang C., Cao Y. (2020). Graphene Oxide Size-Dependently Altered Lipid Profiles in THP-1 Macrophages. Ecotoxicol. Environ. Saf..

[348] Liu D., Wang L., Wang Z., Cuschieri A. (2012). Different Cellular Response Mechanisms Contribute to the Length-Dependent Cytotoxicity of Multi-Walled Carbon Nanotubes. Nanoscale Res. Lett..

[349] Song M., Yuan S., Yin J., Wang X., Meng Z., Wang H., Jiang G. (2012). Size-Dependent Toxicity of Nano-C60 Aggregates: More Sensitive Indication by Apoptosis-Related Bax Translocation in Cultured Human Cells. Environ. Sci. Technol..

