# Supplementary material for: Potential Environmental and Health Implications from the Scaled-Up Production and Disposal of Nanomaterials Used in Biosensors
Source: Biosensors (Basel). 2022 Nov 25;12(12):1082. doi: 10.3390/bios12121082 (PMC9775545; doi:10.3390/bios12121082)
Supplement: Supplementary file 1 [file biosensors-12-01082-s001.zip › biosensors-2043893-supplementary.pdf]

biosensors

**Supplementary Materials:** The following supporting information can be downloaded at: www.mdpi.com/xxx/s1,

**Table S1.** List of commonly used nanomaterials in biosensors development. Each nanoparticle category is associated with a non-comprehensive set of biosensing applications, as well as their reported properties for enhanced device performance, and detection modes.

| Nanomaterial | Biosensing Applications | Reported properties | Operation Modes /References |
|---|---|---|---|
| **Nanometal** | | | |
| Copper | Detection of biological molecules, viruses, pathogens | Low cost and affordable<br>Ease of preparation<br>Optical properties (strong fluorescence, MegaStokes shifting, surface plasmon resonance (SPR))<br>Electrochemical properties (high sensitivity, rapid response), small size, high surface to volume ratio | Fluorescence: [282,283]<br>Electrochemical: [284–286]<br>Colorimetric: [287]<br>References: [288–290] |
| Gold | Detection of pathogens (bacterial zoonosis, viruses, etc.) biological molecules (hormones, etc.), cancer cells, food pathogens (Staphylococcus aureus) | Optical properties (plasmonic resonance, rapid equipment-free detection, etc.)<br>Simple framework<br>Low-cost<br>Chemical and physical stability<br>Biocompatibility<br>Conductivity<br>Water solubility<br>Facile conjugation to biomolecules<br>Sensitive | Electrochemical: [286,291,292]<br>Plasmonic Resonance: [293–295]<br>Colorimetric: [296,297]<br>Fluorescence: [298,299]<br>References: [300–302] |
| Platinum | Detection of medication (heparin, dipyrone), biologically relevant molecules (enzymes, glucose), food pathogens (Staphylococcus aureus) | Ease of preparation<br>Catalytic properties (redox reactions, hybridization)<br>Resistant to corrosion and oxidation<br>High-sensitivity<br>Low-detection limits | Electrochemical: [303–305]<br>Colorimetric: [306–308] |
| Silver | Biologically relevant molecules ($\alpha$-fetoprotein (AFP), C-reactive protein (CRP), dopamine, MicroRNA, etc.); Medication (Ethambutol and Pyrazinamide) | Optical properties (high plasmon resonance in visible and near infrared regions, high brightness, and photostability)<br>Chemical instability and oxidation<br>Ease of preparation of nanoparticles and hybridized composites<br>Easy conversion to silver ions<br>Low cost<br>Abundant | Plasmonic Resonance: [309–311]<br>Fluorescence: [312,313]<br>Electrochemical: [314] |

| | | | |
|---|---|---|---|
| | | Poor adhesion on dielectric surfaces<br>Electrical conductivity<br>Stability<br>Low sintering temperatures | |
| Palladium | Biologically relevant molecules (dopamine) | Effective catalytic properties<br>High affinity towards H2<br>Reversibility | Electrochemical: [315,316]<br><br>Optical: [317]<br><br>References: [318,319] |
| **Nanometal Oxide** | | | |
| Copper Oxide | Biologically relevant molecules (glucose, HPV16 DNA), Fungi (*Aspergillus niger*) | Semiconductor metal oxide<br>Ease of preparation<br>Catalytic properties<br>Antimicrobials<br>Cytotoxic to cancer cell lines<br>Cost-effective dye-degraders | Electrochemical: [320–322]<br><br>Colorimetric: [323]<br><br>References: [324–326] |
| Iron Oxide(s) | Food pathogens (Salmonella); Clinically relevant pathogens *Serratia marcescens*); Enzymes (arginase) | Unique magnetic properties (response to an externally applied magnetic field, Superparamagnetic)<br>Biocompatible and biodegradability<br>Facile to functionalize | Low field nuclear magnetic resonance: [327]<br><br>Electrochemical: [328,329]<br><br>Fluoresces: [330]<br><br>References: [325,331] |
| Titanium Dioxide | Bacteria (E. coli O157:H7), biological temperature, biologically relevant molecules (HPV16 DNA) | Biocompatibility<br>High conductivity<br>Low cost | Electrical: [332]<br><br>Electrochemical: [333]<br><br>Optical: [334]<br><br>References: [325] |
| Zinc oxide | Biologically relevant molecules (HPV16 DNA); Viruses (SARS-CoV-2) | Wide band gap<br>Binding energy<br>Biocompatibility<br>low-cost<br>Stability<br>Semiconducting material<br>Relatively high refractive index<br>Biocompatibility<br>Electrostatic affinity towards biomolecules | Electrochemical: [292,335,336]<br><br>References: [325,337] |

**Table S2.** Studies on the toxicity of 0-D, 1-D, 2-D, and 3-D nanocarbon materials in zebra fish (*Danio rerio*) embryos.

| **Material** | **Concentration (μg/L)** | **Endpoint** | **Reported outcomes** | **Ref** |
|---|---|---|---|---|
| Fullerenes (0-D) | 100 to 500 | Cellular Death | • Cell death (concentration dependent) significant at concentrations greater than or equal to 100 μg/L<br>• 100 percent mortality at 200 μg/L exposure | [338] |
| Multi-walled carbon nanotubes (MWCNTs) (1-D) | 0, 3400, 7600, 12500, 25,000 and 50,000 | Cellular Death; hatching | • Increase in overall cellular apoptosis (concentration-dependent) significant at 50000 μg/L<br>• Significantly delayed hatching | [339] |
| Graphene oxide (GO) sheets (2-D) | 0, 3400, 7600, 12500, 25,000 and 50,000 | Cellular Death; hatching | • Cellular apoptosis (forehead and eye region)<br>• Minor cell growth inhibition<br>• Slight hatching delay (50000 μg/L) | [339] |
| Laser-induced graphene (3-D) | 5000, 10000, 50000 and 100000 | Growth; hatching; embryo development | • Biocompatibility<br>• No toxic effects<br>• No effect on development | [340] |

**Table S3.** Studies on the size-dependent toxicity of nanometal, nanometal oxide, and nanocarbon materials used in biosensing. Studies, where smaller-sized nanomaterials induced higher toxicity, are labeled ↓ size = ↑ toxicity while studies, where larger-sized materials induce higher toxicity, are labeled ↑ size = ↑ toxicity.

| Nanomaterial | Nanoparticle Parameters | Size | Model Organism | End point and results | Reference |
|---|---|---|---|---|---|
| **Nanometal** | | | | | |
| Copper | Shape: Spherical<br>Zeta Potential: Not Reported<br>Surface Functionalization: Not Reported | 30 nm<br>50 nm<br>80 nm<br>1 µm | Male Sprague Dawley rats | Acute (LD50):<br>↓ size = ↑ toxicity<br><br>Short Term:<br>↑ size = ↑ toxicity | [182] |
| Gold | Shape: Spherical<br>Zeta Potential: 5-nm (−10.33 mV), 20-nm (−4.58 mV) and 50-nm (−12.32 mV) | 5 nm<br>20 nm<br>50 nm | HepG2 cells and L02 cells<br>BALB/c mice | Cytotoxicity assay:<br>↓ size = ↑ toxicity<br><br>Hemolysis test: No significant differences<br><br>ROS Generation:<br>↓ size = ↑ toxicity<br><br>Cytokines release:<br>↓ size = ↑ toxicity<br><br>Western blot analysis: ↓ size = ↑ toxicity | [185] |

| | | | | | |
|---|---|---|---|---|---|
| Platinum | Shape: Spherical<br>Zeta Potential: 5 nm (–54.20±0.44 to –11.40±0.40 mV) and 70 nm (–39.43±0.62 to –10.04±0.17 mV) | 5 nm<br>70 nm | Neonatal mice ventricular cardiomyocytes | Cardiomyocyte function: No significant differences<br><br>ROS Generation: No significant differences<br><br>Extracellular lactic dehydrogenase (LDH): No significant differences | [37] |
| Silver | Shape: Spherical<br>Zeta Potential: Not reported | 10 nm<br>60 nm | Human hepatocarcinoma cells (HepG2) | Cell Viability Assay:<br>↓ size = ↑ toxicity | [341] |
| Palladium | Shape: Spherical<br>Zeta Potential: Not reported | 2.0±0.1 nm<br>2.5±0.2 nm and 3.1±0.2 nm | Gram negative *E. coli*<br><br>Gram positive *S. aureus* | Inhibitory growth effects *E. coli*:<br>↓ size = ↑ toxicity<br><br>Inhibitory growth effects *S. aureus:* mid-sized (2.5 nm) particles most toxic and smaller (2.0 nm) and larger (3.1 nm) less toxic | [197] |
| **Nanometal Oxide** | | | | | |
| Copper Oxide | Shape: Not reported<br>Zeta Potential: Not reported | 4 nm<br>24 nm | A549 human lung cell line | Cell toxicity assay:<br>↑ size = ↑ toxicity | [342] |
| Iron Oxide(s) | Shape: Spherical<br>Zeta Potential: −3.99 ± 0.70 mV and −12.43 ± 1.21 mV | 10 nm<br>30 nm | Bone marrow cells Female C57BL/6 mice | mRNA transcription of inflammation-related genes in macrophages:<br>↑ size = ↑ toxicity | [343] |
| Titanium Dioxide | Shape: Square<br>Zeta Potential: Not Reported | 100 nm<br>50 nm<br>30 nm<br>10 nm | Human umbilical vein endothelial cells (HUVECs) | Comet assay (DNA damage):<br>↓ size = ↑ toxicity<br><br>Micronucleus test:<br>↓ size = ↑ toxicity<br><br>ROS Generation:<br>↓ size = ↑ toxicity<br><br>Glutathione (GSH) assay: ↑ size = ↑ toxicity (antioxidative ability)<br><br>Western Blot (levels of Nrf2 protein):<br>↓ size = ↑ toxicity | [344] |
| Zinc oxide | Shape: Spherical<br>Zeta Potential: -10.3 and -10.0 mV | 50 nm<br>200 nm | Human lung adenocarcinoma A549 cell line | Cell viability (MTS assay):<br>↓ size = ↑ toxicity | [345] |

| | | | | | |
|---|---|---|---|---|---|
| | | | | Mitochondrial damage and oxidative stress:<br>↓ size = ↑ toxicity<br><br>Autophagic process:<br>↓ size = ↑ toxicity<br><br>Autophagic dysfunction:<br>↓ size = ↑ toxicity<br><br>Lysosomal function:<br>↓ size = ↑ toxicity | |
| **Carbon** | | | | | |
| Graphene | Shape: Not Reported<br>Zeta Potential: Not Reported | S-G (avg 29.31 nm)<br>M-G (307.56 nm)<br>L-G (not reported) | Zebrafish (Danio rerio)<br>HEK 293T cells | Cell viability: Toxicity S-G < L-G < M-G<br><br>DNA damage (HEK 293T): Toxicity M-G < L-G < S-G<br><br>Hatching rate: S-G < M-G <L-G<br><br>Body length: M-G < S-G < L-G<br><br>Body weight: S-G < M-G and L-G<br><br>ROS Generation (Zebrafish): S-G < M-G < L-G | [346] |
| Graphene oxide (GO) | Shape: Not Reported<br>Zeta Potential: GO- L > GO-S | GO-Large (thickness 0.8–1.2 nm, size 500–5000 nm)<br>GO-Small (thickness 0.8–1.2 nm, size < 500 nm | Macrophages (THP-1 monocytes) | Cytotoxicity:<br>↑ size = ↑ toxicity | [347] |
| Multi-walled carbon nanotubes | Shape: Nanotube<br>Zeta Potential: Not reported | Diameter (constant): 40 to 60 nm<br><br>Length (Varied): 3 to 14 μm or 1.5 μm | RAW264.7 cells<br><br>MCF-7 cells | Cell viability assays: ↑ Length = ↑ toxicity | [348] |
| Fullerenes (buckyball) | Shape:<br>Small: smooth and round | Hydrodynamic diameter: | GFP-Bax stable MCF7 cells | Cytotoxicity:<br>↓ size = ↑ toxicity | [349] |

| | | | | |
|---|---|---|---|---|
| | Larger: amorphous and angula<br>Zeta Potential: Not reported | 108.6 ± 3.5 to 228.2 ± 7.5 | A549 cells | Bax translocation:<br>↓ size = ↑ toxicity |
